# Understanding the molecular mechanism of pathogenic variants of BIR2 domain in XIAP-deficient inflammatory bowel disease

**DOI:** 10.1038/s41598-023-50932-5

**Published:** 2024-01-09

**Authors:** Juhwan Lee, Kyoung Mi Sim, Mooseok Kang, Hyun Ju Oh, Ho Jung Choi, Yeong Eun Kim, Chan-Gi Pack, Kyunggon Kim, Kyung Mo Kim, Seak Hee Oh, Inki Kim, Iksoo Chang

**Affiliations:** 1 iProtein Therapeutics Inc., Munji-ro 281-9, Yuseong-gu, Daejeon, Korea; 2 Department of Convergence Medicine, Asan Medical Center, Asan Institutes for Life Sciences, University of Ulsan College of Medicine, Seoul, Korea; 3 Department of Pediatrics, Asan Medical Center Children’s Hospital, University of Ulsan College of Medicine, 88, Olympic-ro 43-gil, Songpa-Gu, Seoul, 05505 Korea; 4 Department of Pharmacology, University of Ulsan College of Medicine, 88, Olympic-ro 43-gil, Songpa-Gu, Seoul, 05505 Korea; 5 Creative Research Initiatives Center for Proteome Biophysics, Department of Brain Sciences and Supercomputing Bigdata Center, DGIST, Daegu, 42988 Korea; 6 Department of Brain Sciences and Supercomputing Big Data Center, DGIST, Daegu, 42988 Korea

**Keywords:** Molecular biology, Structural biology

## Abstract

X-linked inhibitor of apoptosis protein (XIAP) deficiency causes refractory inflammatory bowel disease. The XIAP protein plays a pivotal role in the pro-inflammatory response through the nucleotide-binding oligomerization domain-containing signaling pathway that is important in mucosal homeostasis. We analyzed the molecular mechanism of non-synonymous pathogenic variants (PVs) of XIAP BIR2 domain. We generated N-terminally green fluorescent protein-tagged XIAP constructs of representative non-synonymous PVs. Co-immunoprecipitation and fluorescence cross-correlation spectroscopy showed that wild-type XIAP and RIP2 preferentially interacted in live cells, whereas all non-synonymous PV XIAPs failed to interact properly with RIP2. Structural analysis showed that various structural changes by mutations, such as hydrophobic core collapse, Zn-finger loss, and spatial rearrangement, destabilized the two loop structures (174–182 and 205–215) that critically interact with RIP2. Subsequently, it caused a failure of RIP2 ubiquitination and loss of protein deficiency by the auto-ubiquitination of all XIAP mutants. These findings could enhance our understanding of the role of XIAP mutations in XIAP-deficient inflammatory bowel disease and may benefit future therapeutic strategies.

## Introduction

X-linked inhibitor of apoptosis protein (XIAP) deficiency (OMIM #300635) is one of the main causes of X-linked lymphoproliferative syndrome and monogenic inflammatory bowel disease (IBD)^1,2^. *XIAP* is located on the X-chromosome and a single *XIAP* mutant allele in a male patient (hemizygous) causes these diseases. XIAP contains baculovirus IAP repeat (BIR) 1, BIR2, BIR3, ubiquitin-associated (UBA), and really interesting new gene (RING) domains^3^. The RING domain has E3 ubiquitin–protein ligase that ubiquitinates XIAP domain-bound proteins. XIAP plays multifunctional roles mainly in cell apoptosis and inflammatory response to bacterial pathogens by directly interacting with various intracellular proteins. First, XIAP directly inhibits activated caspases to protect cells from undergoing apoptosis in response to a diverse set of cellular stresses in activated immune cells, particularly T cells^4^. Defective XIAP in patient peripheral blood mononuclear cells caused activation-induced cell death in various in vitro experiments^1,2^. Second, XIAP is critically involved in nucleotide-binding oligomerization domain-containing protein (NOD) signaling, which is essential in innate immunity^5^. To activate NOD signaling in response to intracellular bacterial pathogens, such as muramyl dipeptide (MDP), and induce subsequent NF-κB/MAPK pathways, receptor-interacting-serine/threonine-protein kinase 2 (RIP2) requires to be ubiquitinated and XIAP directly interacts with and ubiquitinates RIP2 through its RING domain. Since NOD signaling is critical in IBD development^6,7^, defective interaction of mutant XIAPs with RIP2 and subsequent defective NOD signaling are the core pathogenesis of XIAP-deficient IBD. Indeed, defective NOD signaling was noted among patients with XIAP-deficient IBD^8^ and flow cytometry test for the NOD signaling is an essential functional assay in diagnosing pathogenic variants (PVs) in the *XIAP* gene^9^. Besides these two main roles of XIAP, our understanding of its multifunctional roles are still expanding^10^.

Based on the understanding the core mechanism of XIAP, drug development strategy has been mainly focused on anti-cancer effect by suppressing the activity of XIAP and their related partners^11,12^. In new drug development for IBD, XIAP inhibition has also become a potential therapeutic target that may reduce over-inflammation via apoptosis induction in immune cells and decreased NF-κB activity^3^. However, there is no promising suggestion of a new drug for XIAP deficiency. Currently, hematopoietic stem cell transplantation (HSCT) is the only practical therapeutic option^13^. Beside this extreme treatment measure that may be life-threatening, medical treatments including chemotherapies and biologics showed no or temporal effect in controlling hyperinflammatory conditions and XIAP-deficient colitis^14,15^. Therefore, the analysis of risk and benefit of HSCT is controversial and no solid guideline is available to determine the benefits of HSCT in patients with XIAP deficiency. To find a therapeutic target in XIAP deficiency, understanding the detailed mechanism of XIAP mutants in patients is needed. Based on the molecular explanations about the pathogenicity, we may identify a possible target point and establish an appropriate drug development strategy. Herein, we analyzed the molecular mechanism of pathogenic variants of XIAP BIR2 domain, which is a critical site in controlling NOD signaling.

## Materials and methods

### Selection of pathogenic variant of interest in XIAP

The sequence information for the *XIAP* mutants (reference sequence: NM_001167.3) was obtained from public datasets (NCBI dbSNP, UniProt, and gnomAD). The *XIAP* PVs were reviewed based on previously published studies. The Web of Science, PUBMED, and Cochrane-Library databases were searched for clinical cases of XIAP deficiency in humans. Representative 14 (10 non-synonymous variants and 4 null variants) PVs on BIR2 domain reported in human patients were noted and the 10 non-synonymous PVs were evaluated (Supplementary Fig. 1).

### Mutant XIAP expression constructs

To identify the characteristics of the interaction of XIAP mutants with RIP2 in live HEK 293 T cells, we prepared an N-terminally green fluorescent protein (GFP)-tagged XIAP-WT construct (pEGFP–XIAP-WT). The cDNA clones containing full-length human *RIP2* (KUGI #hMU008516) and *XIAP* (KUGI #hMU005178) coding sequences were purchased from the Korea Human Gene Bank (KHGB). The PCR-amplified full-length *XIAP* open reading frame (ORF) was digested with *Sal*I and *Bam*HI, and subcloned into the *Xho*I and *Bam*HI sites of pEGFP-C2 (Takara Bio, USA). For site-directed XIAP-C203Y, -ΔG204, -R166I, -R166K, -W173G, -G188E, -L189P, -V198M, -L207P, and -H220Y mutagenesis, the whole plasmid from pEGFP–XIAP-WT was PCR-amplified, digested with *Dpn*I to remove the template, and then cloned by self-ligation. To produce the N-terminal red fluorescent protein (RFP)-tagged RIPK2-WT-expressing construct (pmRFP–RIP2-WT), pmRFP-C2, an mRFP expression vector, was generated using the pMRS reporter construct (ToolGen, Seoul, Korea), and the PCR-amplified full-length *RIP2* ORF was subcloned into the *Bgl*II and *Eco*RI sites of pmRFP-C2.

### Cell culture and transfection

HEK 293 T cells (CRL-3216, ATCC, Manassas, VA) were cultured in Dulbecco’s modified Eagle’s medium (DMEM; Cytiva, Massachusetts, USA) supplemented with 10% fetal bovine serum (FBS; Gibco, Waltham, MA, USA), 1% antibiotic penicillin–streptomycin, and 2.05 mM L-glutamine and maintained at 37 °C in a humidified, 5% CO_2_-containing atmosphere. To express the GFP-tagged XIAP WT or mutants, and RFP-tagged RIP2 in 293 T cells, 10^6^ cells/well in six-well plates were transfected with a mixture comprising 8 µL Lipofectamine 2000 (ThermoFisher, Waltham, MA); 1 µg pEGFP, pEGFP–XIAP-WT, pEGFP–XIAP-mutant plasmid; and 3 µg pmRFP–RIP2 plasmid for 6 h. Then, the media were replaced with normal culture media (DMEM supplemented with 10% FBS).

### Co-immunoprecipitation (co-IP)

HEK 293 T cells were transfected with various plasmids using Lipofectamine 2000 for 6 h. Then, the media were replaced with normal culture media. After transfecting for 24 h, the cells were treated with 10 µM MG132 (Enzo, Farmingdale, NY, USA) for 4.5 h before harvesting. Then, the cells were lysed with RIPA buffer (ThermoFisher, Waltham, MA) supplemented with 1 × protease inhibitor (Roche, Basel, Switzerland), 1 × phosphatase inhibitor (Roche, Basel, Switzerland), and 100 µM PMSF (Sigma, Burlington, MA). The lysates were centrifuged at 15,000 rpm at 4 °C for 20 min. Among the same amount of protein in the supernatant by BCA analysis, 10% supernatant was immunoblotted to confirm each protein expression. The remaining supernatants were incubated with 1 mg anti-GFP antibody (Santa Cruz Biotechnology, Dallas, TX, USA) overnight at 4 °C. The antibody-bound proteins were pulled down with 40 µL Protein A/G Plus agarose (Santacruz, CA) for 1 h at 4 °C. The beads were washed three times with RIPA buffer and heated in 1 × SDS sample loading buffer (BioSesang, Seoul, Korea). The eluted proteins were immunoblotted with anti-RFP (ThermoFisher, Waltham, MA, USA), anti-GFP(Santacruz, CA) and anti-β-actin (Novus, Toronto, Canada) antibodies.

### Fluorescence cross-correlation spectroscopy (FCCS)

To quantitate the dynamically coordinated interaction between XIAP mutants and RIP2 in live HEK 293 T cells, FCCS was performed on an LSM780 inverted confocal laser scanning microscope (Carl Zeiss) as described previously^16,17^. The FCCS setup consisted of a continuous-wave Ar^+^ laser (25 mW), a solid-state laser (20 mW), a water-immersion objective (C-Apochromat, 340/1.2 NA; Carl Zeiss), and a GaAsP multichannel spectral detector (Quasar; Carl Zeiss). GFP on XIAP mutants and RFP on RIP2 were excited with 488 and 561 nm laser lines, respectively, with minimal total power to optimize the signal-to-noise ratio. The emission signals were split using a dichroic mirror (488/561 nm beam splitter); GFP and RFP were detected at 500–530 (green channel) and 600–650 (red channel) nm, respectively. All measured fluorescence auto-correlation functions (FAFs) of GFP-tagged XIAP and RFP-tagged RIP2 in live cells were globally fitted using the ZEN 2012 software installed on the LSM780 system using the two-component model (*i* = 2) with or without the triplet term^17^. Finally, the cross-correlation function amplitude was normalized to the auto-correlation function amplitude of GFP or RFP to calculate the relative cross-correlation amplitude (values between 0 and 1) corresponding to the fraction of associated molecules. Relative cross-correlation amplitude close to 1 indicates a high probability of coordinated dynamic interaction between XIAP mutants and RIP2.

### FCCS data analysis

To quantify the FAFs, FCCS data were analyzed using the ZEN 2012 acquisition software (Carl Zeiss). All dual-color FCCS were performed at 25 °C on an LSM780 confocal microscope as described previously^17^. Briefly, the FAFs of the red and green channels, G_*r*_ (τ) and G_*g*_ (τ), respectively, and the fluorescence cross-correlation function (FCF), G_*c*_ (τ), were calculated from

1 $${G}_{x}\left(\tau \right)=1+\frac{\langle {\delta I}_{i}\left(t\right)\cdot {\delta I}_{j}\left(t+\tau \right)\rangle }{\langle {I}_{i}\left(t\right)\rangle \langle {I}_{j}\left(t\right)\rangle }$$

where τ is time delay, *I*_*i*_ is fluorescence intensity of the red (*i* = *r*) or green channel (*i* = *g*), and G_*r*_ (τ), G_*g*_ (τ), and G_*c*_ (τ) denote the red channel FAF (*i* = *j* = *x* = *r*), green channel FAF (*i* = *j* = *x* = *g*), and FCF (*i* = *r*, *j* = *g*, *x* = *r*), respectively. The acquired G_*x*_ (τ) values were fitted using a one-, two-, or three-component model as follows:

2 $${G}_{x}\left(\tau \right)=1+\frac{1}{N}\sum_{i}{F}_{i}{\left(1+\frac{\tau }{{\tau }_{i}}\right)}^{-1}{\left(1+\frac{\tau }{{s}^{2}{\tau }_{i}}\right)}^{-1/2}$$

where *F*_*i*_ and τ_*ι*_ are the fraction and diffusion time of component *I*, respectively; *N* is the average number of fluorescent particles in the excitation-detection volume defined by radius *w*_*0*_ and length 2*z*_*0*_; and *s* is a structural parameter representing the ratio *s* = *z*_*0*_/*w*_*0*_. The structural parameter was calibrated using Rhodamine-6G (Rh6G) solution. Positions for FCCS measurement were selected within the cytosol. All measured FAFs and FCFs from live cells were globally fitted using the software installed on the LSM780 system using the two-component model (*i* = 2) with or without the triplet term. For simplicity, the triplet term in Eq. (2) is not shown. To evaluate the interaction amplitude, the cross-correlation function amplitude was normalized to the auto-correlation function amplitude of GFP or RFP to calculate the relative cross-correlation amplitude (RCA; [G_c_(0) − 1]/[G_r_(0) − 1]) corresponding to the fraction of associated molecules (*N*_c_/*N*_g_). As negative control, FCCS was performed using EGFP and RFP–RIP2 co-expressing cells.

### Ubiquitination assay

For ubiquitination assays, HEK 293 T cells were transfected with the indicated plasmids using Lipofectamine 2000 for 6 h. Then, the media were replaced with normal culture media. After transfecting for 24 h, the cells were treated with 10 µM MG132 for 2 h before harvesting. The cells were then lysed using co-IP lysis buffer supplemented with 20 mM NEM (Sigma, Burlington, MA). The cell lysates were centrifuged at 15,000 rpm at 4 °C for 20 min. Among the same amount of protein in the supernatant by by bicinchoninic acid (BCA) analysis, 10% supernatant was immunoblotted to confirm each protein expression. The remaining supernatants were incubated with 1 mg anti-GFP (Santacruz, CA, USA) or anti-RFP antibodies overnight at 4 °C. The antibody-binding proteins were pulled down by incubation for 1 h at 4 °C with 20 µL Protein A or G magnetic beads (ThermoFisher, Waltham, MA). The beads were washed three times with co-immunoprecipitation lysis buffer and heated in 1 × SDS sample loading buffer. The eluted proteins were immunoblotted with anti-RFP, anti-GFP, anti-ubiquitin (Santacruz, CA), or anti-β-actin antibodies.

### In-gel trypsin digestion

Excised gel pieces from 1DE SDS–PAGE were washed with 500 mL distilled water and the stained Coomassie brilliant blue was removed from the gel with 300 μL 250 mM ammonium bicarbonate, 50% acetonitrile (ACN) for 3 h. After washing with distilled water, the hydrated gel was dehydrated in 100% ACN until they turned opaque and rehydrated with 100 mM ammonium bicarbonate until they turned transparent. This dehydration and rehydration process was repeated three times, followed by a single dehydration in 100% ACN. The gel pieces were dried in a vacuum centrifuge and rehydrated at 47 °C for 45 min using a digestion buffer containing mass grade 0.01 mg/mL trypsin/LysC in 50 mM ammonium bicarbonate (Promega, Madison, WI, USA) and incubated overnight (16 h) at 37 °C. The digested peptide solutions were moved to new tubes, dried, and stored at − 80 °C until mass spectrometry.

### Liquid chromatography-tandem mass spectrometry (LC–MS/MS) and database search

The peptide mixture for each sample set was reconstituted in 0.1% formic acid and the peptides were separated using Ultimate3000 RSLC system coupled with a Q Exactive HFx mass spectrometer (ThermoFisher, Waltham, MA). The liquid chromatography gradient and data-dependent acquisition-MS used were as reported previously^18^. The acquired MS spectra were searched using Sequest HT on Proteome discoverer (version 2.3, ThermoFisher, Waltham, MA) against the SwissProt human proteome sequence database (May 2017) using variable modification at lysine residue with di-glycine. Moreover, label-free quantities of each di-glycine-attached target protein peptide was extracted and further analyzed.

### Temperature-based replica exchange all-atom molecular dynamics (T-REMD) simulation of the 3D structure of XIAP and RIP2 domains

The initial structures of eight BIR2 mutants were generated based on the BIR2-WT domain structure with the TIP3 water model^19^ (PDB code: 8AZA). Na^+^ or Cl^–^ ions were added to neutralize the protein systems, and no more ions were added for ionic concentration. The energy barrier between the local minima is the main hurdle in observing the structural change in the BIR2 mutant domain. Sufficient kinetic energy represented by high temperature is required to overcome the high energy barrier. We used T-REMD simulation to overcome the energy barriers based on system temperature exchanges with AMBER22 simulation package with ZAFF model and ff19SB force field^20,21^. We used a narrow temperature interval between the replicas (approximately 3.7 K) to sufficiently exchange the system temperature. The cutoff for short-range non-bonded interactions was 9 Å and the particle-mesh Ewald method was applied for long-range electrostatic interactions^22^. The temperature was regulated by Langevin dynamics with γ = 2.0 collision frequency^23,24^. The SHAKE algorithm^25^ was used for the hydrogen atoms bond length. Prior to the production run, 2000 steps of steepest-descent minimization followed by 2000 steps of conjugate gradient minimization, a 100 ps heating process with a 15.0 kcal/molÅ^2^ restrain on the protein backbone, and a 1 ns side-chain equilibration with a 0.5 kcal/molÅ^2^ restrain on the protein backbone was performed. In the NPT ensemble, the T-REMD production run was performed with eight replicas at 300.00, 303.72, 307.48, 311.26, 315.08, 318.93, 322.82, and 325.00 K^26^. The pressure is regulated to 1 atm using a weak-coupling barostat with 10 ps time constant. Thus, the replica exchanges were attempted every 10 ps. During the 2 μs simulation run time, all replicas attempted 200,000 exchanges, completing an average of 18,000 successful exchanges, with an approximate 9% exchange probability. The cumulative simulation time was 144 μs (2 μs for each eight replicas and nine protein systems) with 2 fs simulation time step. The final 1.0 μs ensemble for each replica was used for statistical analysis. The CPPTRAJ toolset in the AmberTools20 package^27^ was used for the basic calculations and all the protein structures were created using VMD software in 300 K ensembles^28^. In the case of hydrogen bonding, the angle cut-off is 135° and the distance cut-off is 3.6 Å between the donor atom (H) and the acceptor atoms (C, O, N, F, and S). The initial backbone structure is used as a reference structure in the structural alignment for the root mean square deviation (RMSD) calculations. Additional data including simulation input parameter files and starting structures are available at the following link.

(https://drive.google.com/drive/folders/18jwGrfkIcutMEJ1aKRsMsPqOyrkcGQVL?usp=sharing).

## Results

### BIR2-mutant XIAPs failed to interact with RIP2 in vitro

Various studies have demonstrated that the BIR2 domain of XIAP is recruited to the NOD2 signaling complex through RIP2, excluding the involvement of other XIAP domains (Supplementary Fig. 1)^1,5,6,29^. Damgaard et al. have reported that some non-synonymous XIAP BIR2 mutations abolished the binding with RIP2^5^. We also focused on the specific impact of all non-synonymous PVs on XIAP BIR2 function. Accordingly, we examined the interaction between XIAP and RIP2 in a mammalian system by constructing GFP-tagged WT (XIAP-WT) or 10 mutant XIAPs, and RFP-tagged RIP2-WT vectors and co-expressing them in HEK 293 T cells.

**Figure 1:**
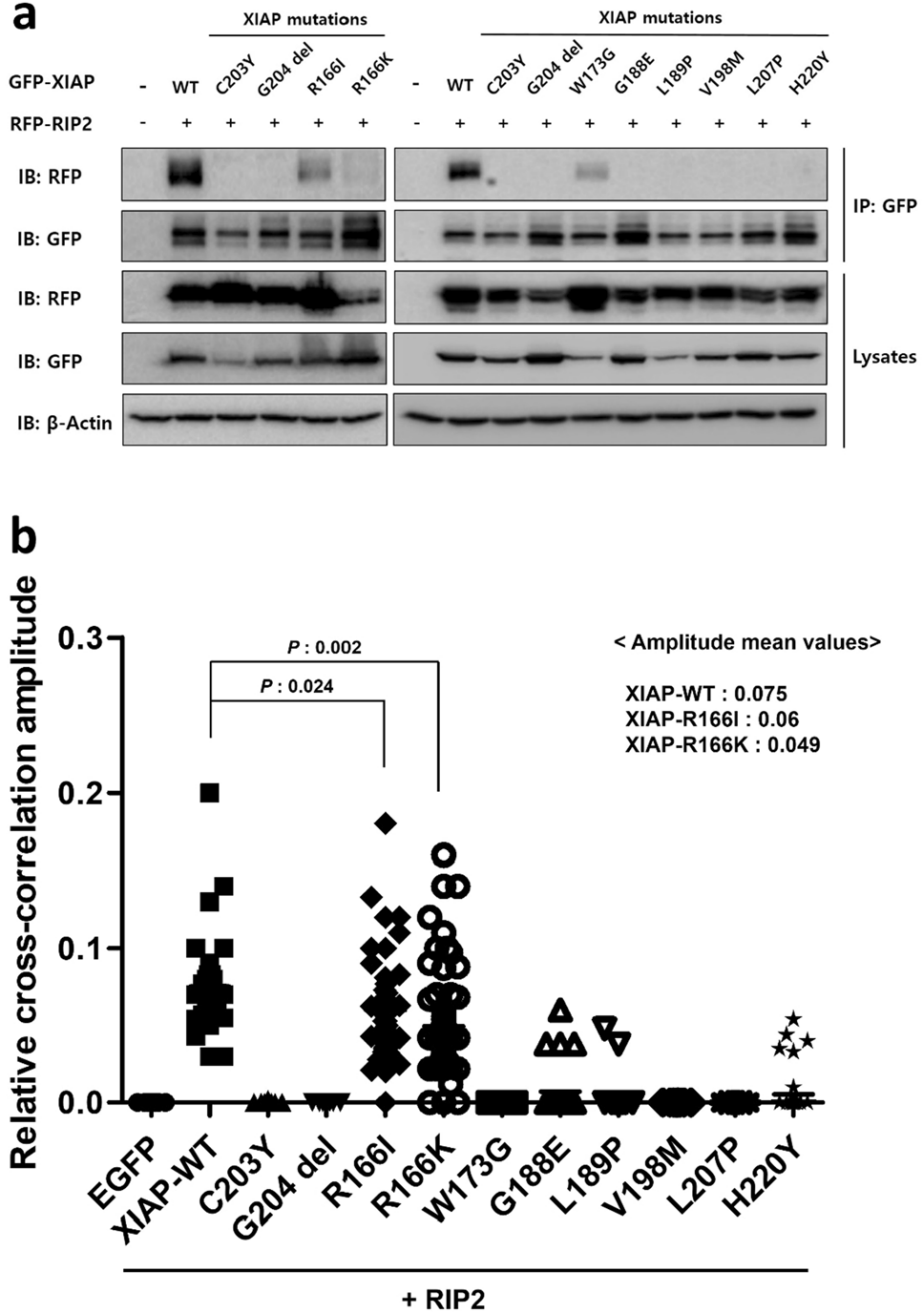
Mutant XIAP BIR2 with non-synonymous PVs failed to interact with RIP2. (**a**) Co-IP of GFP-tagged XIAP WT or mutants, and RFP-tagged RIP2 confirmed their interaction. The tagged XIAP was immunoprecipitated from lysates of HEK 293 T cells transfected with plasmids encoding the indicated GFP-tagged XIAP WT or mutants, and western blot images were cropped for clarification. (**b**) Comparison of relative correlation amplitudes of live HEK 293 T cells expressing the indicated XIAP mutants. The relative correlation amplitudes between GFP–XIAP mutants and RFP-RIP2 were determined by confocal imaging during FCCS. The negative control indicates a mean value of relative correlation amplitude of 0, obtained from cells co-expressing EGFP and RIP2. The mean amplitudes of XIAP mutants were significantly lower than that of the positive control (XIAP-WT + RIP2).

Co-IP experiments were conducted using anti-GFP and anti-RFP antibodies for immunoprecipitation and immunoblotting, respectively. We have previously showed that the interaction between GFP–XIAP-ΔG204 (deletion of G204) and RIP2 protein was significantly weaker than between XIAP-WT and RIP2^8^ and the molecular mechanism of XIAP-ΔG204 was similar to that of XIAP-C203Y, which was Zn-finger loss. We then used GFP–XIAP-WT as a positive control and GFP–XIAP-ΔG204 and GFP–XIAP-C203Y as negative controls to compare the mechanisms of XIAP-R166I, -R166K, -W173G, -G188E, -L189P, -V198M, -L207P, and -H220Y. Co-IP using positive and negative controls confirmed that XIAP-WT was strongly associated with RIP2, whereas XIAP-ΔG204 and -C203Y were not (Fig. 1a). The RFP immunoblot signals in a GFP-immunoprecipitated HEK 293 T cell sample transfected with XIAP-WT were strong, suggesting that XIAP-WT binds to RIP2. However, no protein signals were detected in GFP-immunoprecipitated samples with XIAP-ΔG204 and -C203Y, suggesting less binding with RIP2. Thus, detecting no or weak RFP signals in GFP-immunoprecipitated samples for testing XIAPs mutant indicate reduced protein–protein interaction between mutant XIAPs and RIP2. Thus, XIAP-G188E, -L189P, -V198M, -L207P, -H220Y did not bind with RIP2, while XIAP-R166I, -R166K, and -W173G partially bonded with RIP2.

We then detected the interaction of XIAP mutants and RIP2 in live cells using FCCS, which is a technique for measuring the kinetics of molecular interactions between proteins at a specific position in single live cells using two spectrally distinct fluorophores^16,17^. When two different proteins form a complex, signals of two proteins such as GFP and RFP co-diffuse as they transit the detection volume (Fig. 1b). The strength of the interaction between the two proteins is quantified as the values of relative cross-correlation amplitude. Similar to co-IP, we co-transfected 293 T cells with plasmids encoding RFP–RIP2 and GFP-tagged XIAP WT or mutants. In addition to the negative controls C203Y and G204del, plasmids encoding RFP–RIP2 and GFP with no XIAP were co-transfected into HEK 293 T cells as another negative control. As expected, the overall relative interaction amplitudes of most XIAP mutants were lower than that of XIAP-WT (Fig. 1b). Specifically, we observed that the interactions of RIP2 with XIAP-W173G, -G188E, -L189P, -V198M, -L207P, and -H220Y were significantly weaker than that with the XIAP-WT. Exceptionally, the interaction of RIP2 with XIAP-R166I and -R166K was also weaker than that with XIAP-WT, but stronger than those with other XIAP mutants, which were similar with the results to the co-IP analysis. Taken together, both co-IP and FCCS showed that XIAP-WT and RIP2 preferentially interacted in HEK 293 T cells. However, while there might be subtle variations, all testing non-synonymous XIAPs PV did not interact with RIP2.

### Structural analysis: hydrophobic cores in XIAP BIR2 mutants

**Figure 2:**
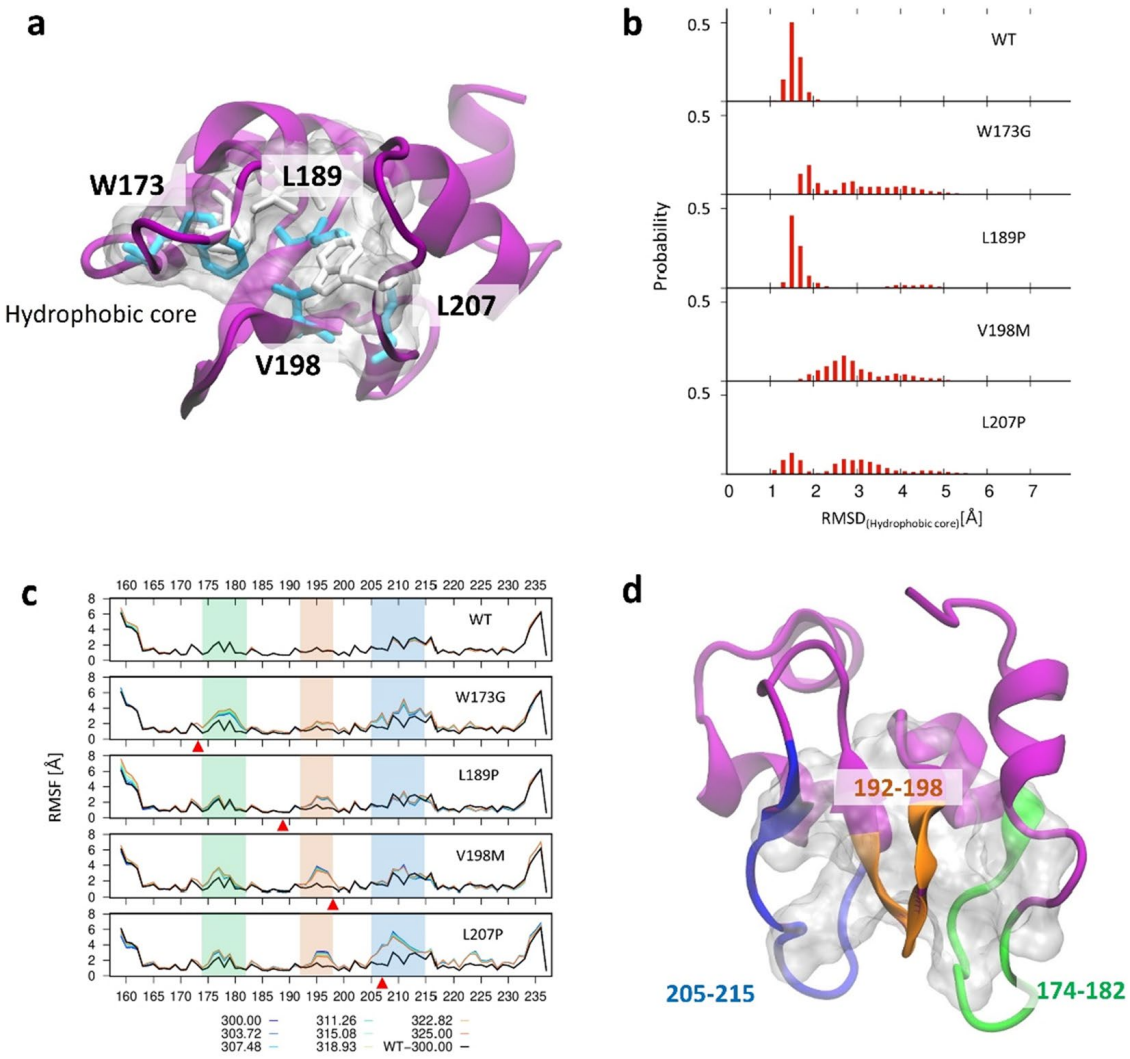
Effect of W173G, L189P, V198M, and L207P mutation on the hydrophobic core. (**a**) Major hydrophobic core of BIR2 domain of XIAP and hydrophobic amino acids members (BIR2: purple cartoon, Hydrophobic core: white transparent surface, Mutated amino acids: cyan sticks, other amino acids: white sticks) (**b**) The root mean square deviation (RMSD) of BIR2- WT and mutants hydrophobic cores. (**c**) The root mean square fluctuation (RMSF) of BIR2- WT and mutants. More fluctuated regions than WT are highlighted in green, orange, and blue. (**d**) XIAP-BIR2 cartoon structure (purple) and fluctuated regions (green, orange, yellow) by mutations.

**Figure 3:**
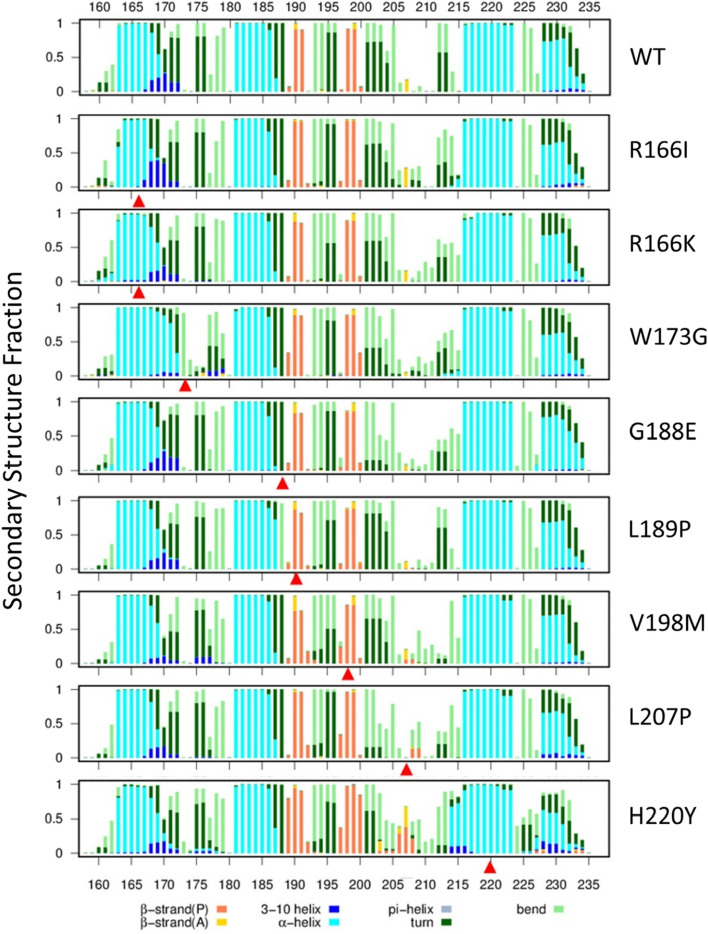
Secondary structure fraction of WT and mutant BIR2 of XIAP protein. Secondary structure fraction of WT and eight mutants over the amino acid count. Mutation points are pointed using red arrows.

To understand the molecular mechanism of weak interaction of XIAP mutants with RIP2, we analyzed each mutant protein structure. We initially focused on an intra-structural hydrophobic interaction inside XIAP that is one of the main driving forces involved in protein folding and substantially affects structural stability and solubility^30^. Accordingly, we noticed that 11 hydrophobic amino acids (F170, W173, A177, L179, L184, A187, L189, V198, L207, W210, and A216) of the BIR2 form the hydrophobic packing on the protein core (Fig. 2a). XIAP-W173G, -L189P, -V198M, and -L207P mutants destabilize this hydrophobic pocket, which changes the BIR2 mutant domain structure (Fig. 2a). We measured the quantitative effect of the mutation on the hydrophobic core by RMSD of the 11 hydrophobic core members. The hydrophobic core of BIR2-WT is stably packed, with 1–2 Å RMSD (WT in Fig. 2b). However, the hydrophobic core is relaxed in four mutant proteins. XIAP-W173G, -V198M, and -L207P show a more dispersed distribution owing to their high RMSD (W173G, V198M, and L207P in Fig. 2b), and XIAP-L189P also has ensembles at 3.5–5 Å, which are not observed in XIAP-WT (L198P in Fig. 2b). The propagation of structural instability due to mutation was also observed while evaluating the root mean square fluctuation (RMSF) in each mutant protein. The red arrow in Fig. 2c indicates the mutated amino acid position. The structural fluctuation increased at 174–182, 192–198, and 205–215 regions in XIAP-W173G, -V198M, and -L207P, respectively. The structural fluctuation of XIAP-L189P increased only in the 192–198 region. Structural images for all three regions are presented in Fig. 2D. Most of the three sections, which are increased fluctuation, consist of loop, bend, or turn secondary structure (Fig. 3). Particularly, the two BIR2 loops at 174–182 and 205–215 regions play a major role in binding to RIP2, according to the recently discovered cryo–EM structure^31^. The first loop (174–182) interacts with RIP2 residues R36 and R41, which are crucial for BIR2–RIP2 binding^32^. Moreover, K209 and I212 of RIP2 interact with the second loop (205–215) structure and are also crucial for BIR2–RIP2 binding in a previous study^33^.

### Structural analysis: contact of two helices in XIAP BIR2 mutants

**Figure 4:**
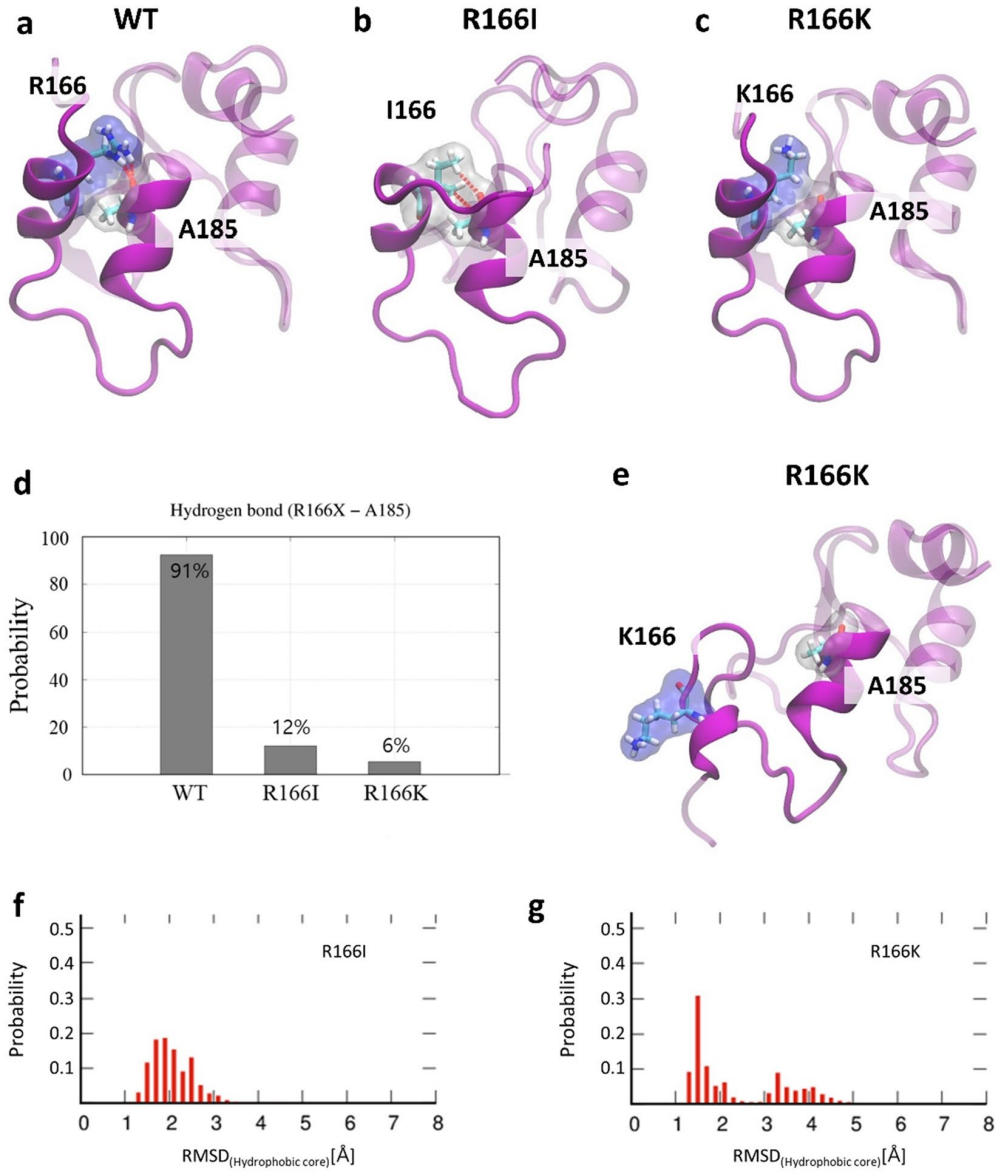
Effect of R166I and R166K mutations on the two alpha-helices. (**a**) Representative structure of WT BIR2, R166, and A185 form a strong hydrogen bond. (**b**) Representative structure of BIR2-R166I mutant. I166 and A185 form a weak hydrogen bond. (**c**) Representative structure of BIR2-R166K mutant. K166 and A185 form a weak hydrogen bond. (**d**) Hydrogen bond formation probability between amino acid on 166 and A185. (**e**) Structural change of R166K between two helices. (**f**) Root mean square deviation (RMSD) of BIR2-R116I hydrophobic core. (**g**) The RMSD of BIR2-R166K hydrophobic core.

Our second interest is changes of hydrogen bonds in R166I and R166K mutant proteins. Hydrogen bonding is one of the most crucial interactions for forming secondary and tertiary protein structures^34^. To form hydrogen bonds appropriately within a protein, the proper distance between two amino acids involved in the hydrogen bonding, as well as the side-chain direction and conformation should be considered. The first (163–168) and second (181–186) helices of BIR2 are in stable contact with hydrogen bonds. In XIAP-R166I and -R166K mutants, R166 and A185 play an important role in developing one of these important hydrogen bonds and maintaining a stable contact (Fig. 4a–c). This hydrogen bond exhibited a 91% stable configuration while simulating BIR2-WT (Fig. 4d). However, owing to the two mutations from arginine (R) to lysine (K) and isoleucine (I), the hydrogen bond is poorly formed because of changes in atomic configuration at the 166th position (R166K: 6%, R166I: 12%). This change in the hydrogen bond weakens the contact between the two helices and may destabilize the whole BIR2 domain tertiary structure. In the XIAP-R166K mutant, the arginine (R) and lysine (K) have side chains with similar characteristics, as they are positively charged. However, K166 cannot form a hydrogen bond with A185. In addition, the K166 not only weakened the contacts between the two helices, but also increased the distance between the two helices (Fig. 4e). In XIAP-R166I mutants, the distance between the two helices did not increase. When the distance between the two helices increases, the structure of the amino acids located in the loop/turn (169–174) connecting the two helices undergo rearrangement; F170 and W173, which were previously mentioned as hydrophobic core members (Fig. 2), are also affected. Subsequently, the hydrophobic core stability is reduced, increasing the RMSD of the hydrophobic core than that of the WT (Fig. 4f and g). Interestingly, as the probability of hydrogen bond formation decreased in XIAP-R166K and -R166I, the relative cross-correlation amplitude of XIAP with RIP2 in FCCS experiments correspondingly reduced.

### Structural analysis: spatial rearrangement by G188E mutation

**Figure 5:**
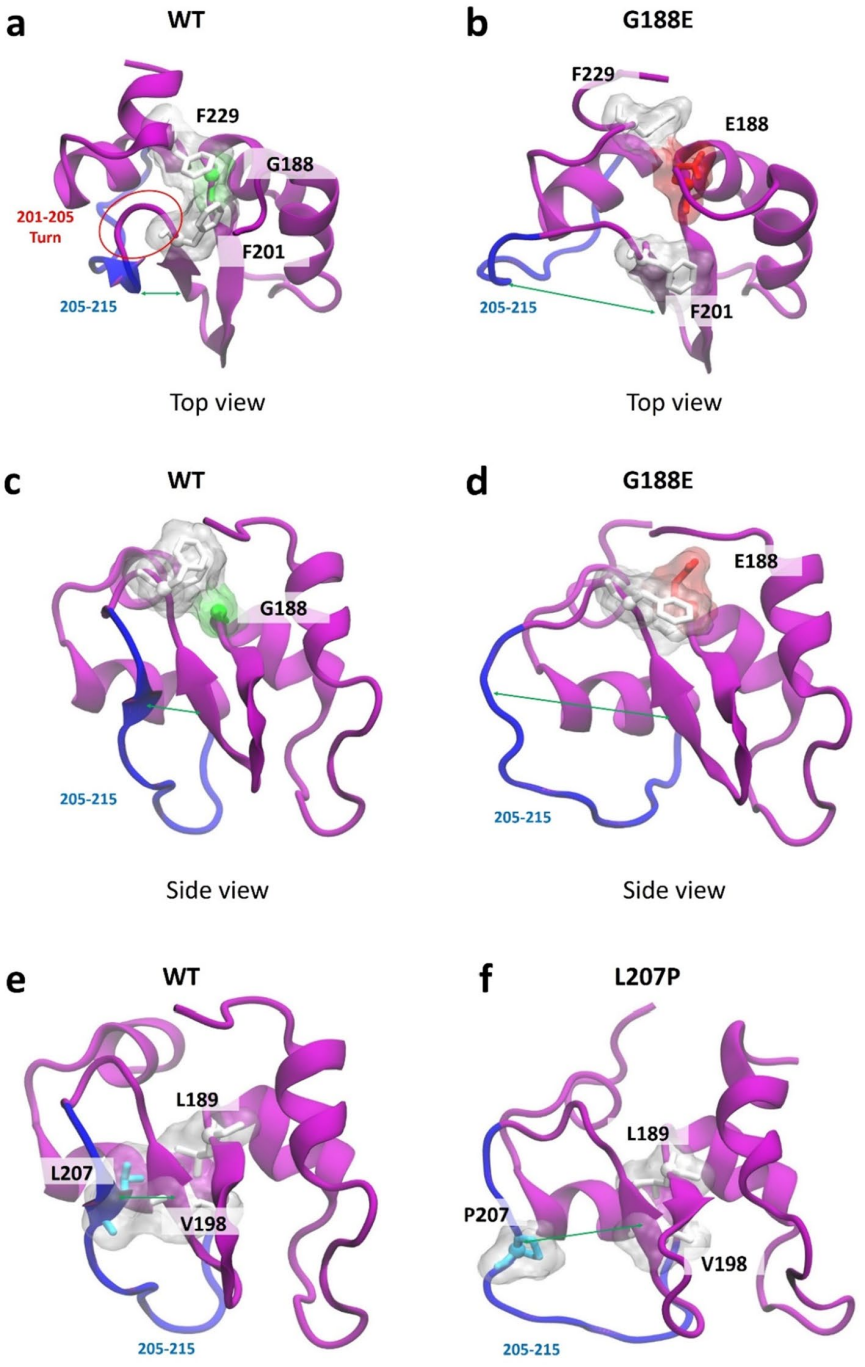
Effect of G188E and L207P mutations on beta structure. (**a**) BIR2-WT structure. Red circle indicates a turn structure between two beta structures. (**b**) BIR2-G188E structure. (**c**) BIR2-WT structure from a different angle. (**d**) BIR2-G188E structure from a different angle. (**e**) BIR2-WT structure from a different angle. (**f**) BIR2-L207P structure from a different angle. Two beta structures were also separated by L207P mutation.

A significant change in the size or characteristic of the side-chain owing to point mutation can alter the interactions between neighboring amino acids, leading to amino acid spatial rearrangement^35^. This occasionally affects the secondary and tertiary structures of the whole protein. In XIAP-G188E mutants, glycine (G) has no side-chain, whereas glutamic acid (E) has a large side-chain with negative polarity, possibly inducing spatial rearrangement of neighboring amino acids. Furthermore, G188 is buried among hydrophobic amino acids, such as F201 and F229 (Fig. 5a). When mutated to E188, the previously covered hydrophobic amino acids F201 and F229 are spatially pushed aside, as the side-chain of glutamic acid tends to be exposed outward (Fig. 5b). First, as F201 is pushed back, the amino acids 201–204, which form a turn structure by forming back-bond hydrogen bonds with each other in the WT, break the hydrogen bonds in the XIAP-G188E mutant and adopt a bent structure (Fig. 3). Consequently, the constraint on the rotation angle is weakened. Accordingly, the distance between two beta structures (198–200 and 206–208) increases (Fig. 5c and Supplementary Fig. 2a, 2b), disruptively widening the 205–215 loop (Fig. 5d and Supplementary Fig. 2c, 2d). Second, XIAP-V198 and -L207 are hydrophobic core members (Fig. 2) on these beta structures, and in close contact with XIAP-L189 (Fig. 5e). However, due to the spatial rearrangement by G188E mutation, the distance between L207 and V198 increases, causing a similar structural change (hydrophobic collapse) after L207P mutation (Fig. 5e, 5f and Supplementary Fig. 2e, 2f.).

### Structural analysis: Zn-finger loss

Our last interest is a Zn-finger structure inside of BIR2 domain, which is already highlighted as a core component in the stability of BIR2, a metalloprotein^8,36,37^. Many experimental and theoretical works have focused on understanding the role of Zn-finger structural stability and folding pathway in metalloproteins^38–42^. Zn-finger plays a crucial role in the stability of the secondary and tertiary structure of the protein, thus impacting the entire folding process. C200, C203, H220, and C227 amino acids in the XIAP BIR2 domain constitute a Zn-finger (holding zinc in covalent bonds of the CCHC pocket) (Supplementary Fig. 3a). Owing to this Zn-finger, a spatial restriction inevitably brings the four CCHC amino acids in close contact. Due to this spatial restriction, the beta structure near C203 and the helix structure near H220 is tightly packed, stabilizing the loop region (205–215) between C203 and H220. This loop region overlaps with the XIAP binding motif-binding pocket and BIR2 binding site (Supplementary Fig. 3a).

Currently, three mutations have been reported on the Zn-finger of BIR2: C203Y, G204del, and H220Y^3,8,43^. We did not investigate C203Y and G204del mutations in this study as we had investigated these mutant structures have been investigated previously^8^. In H220Y mutation, the structural change, loss of this Zn-finger, causes overall structural instability, starting with the beta structure near C203 and the helix near Y220, which lifts the spatial restrictions of C200, C203, Y220, and C227 (Supplementary Fig. 3b). Due to this structural instability, the RMSF of all amino acids constituting BIR2 increased substantially (Supplementary Fig. 3c), and the RMSD of the hydrophobic core also increased the most among the eight pathogenic mutations (Supplementary Fig. 3d). In addition, this change also elevated the RMSF on the two regions of the important BIR2 loops (Fig. 2). Taken together, as expected, the BIR2-H220Y causes Zn-finger loss. Thus, co-IP and FCCS analyses suggested that BIR2-H220Y did not bind to RIP2, similar to BIR2-C203Y and BIR2-ΔG204 with defective Zn-fingers^8^.

### Mutant proteins reduced RIP2 ubiquitination and XIAP auto-ubiquitination

**Figure 6:**
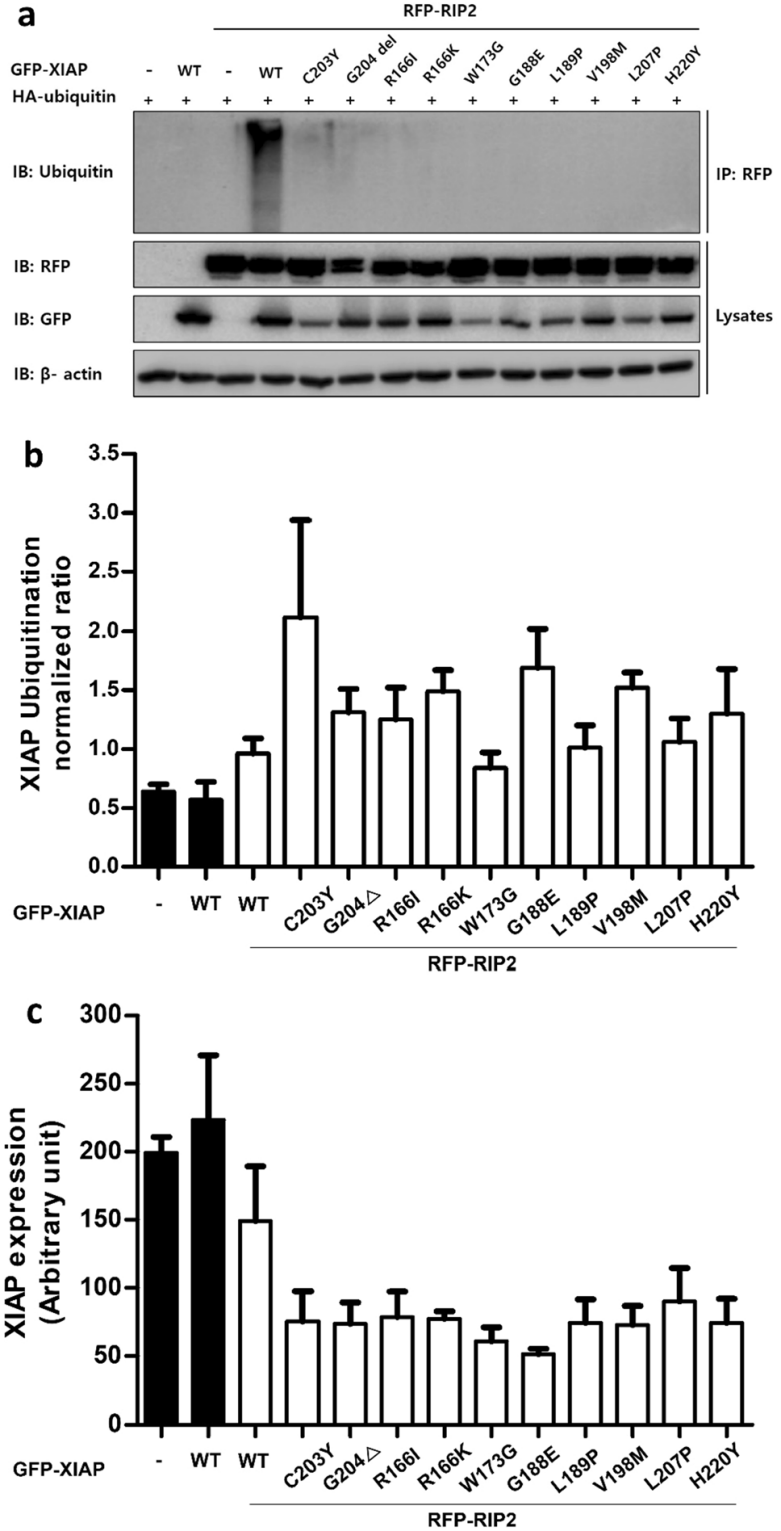
Analysis of XIAP-mediated ubiquitination of XIAP and RIP2. (**a**) Ub conjugates were purified with Protein A or G magnetic beads from lysates of HEK 293 T cells transfected as indicated and examined by immunoblotting. RIP2 ubiquitination was defective in HEK 293 T cells for all XIAP mutants. (**b**) XIAP expression was analyzed in HEK 293 T cells transfected GFP-tagged XIAP WT or mutant constructs, along with RFP-tagged RIP2 by LC–MS. XIAP mutants expression levels were significantly lower compared to XIAP WT expression level. (**c**) XIAP ubiquitination was analyzed in HEK 293 T cells transfected with GFP-tagged XIAP WT or mutant constructs, along with RFP-tagged RIP2 by MS. Auto-ubiquitination of XIAP mutants were significantly higher compared to that of XIAP WT.

Based on our evaluations above, we confirmed that known XIAPs PV are not likely to bind to RIP2 with proper structural explanations using quantitative metrics. XIAP binds to and ubiquitinates RIP2 and if XIAP does not bind to RIP2, XIAP cannot facilitate RIP2 ubiquitination^29,44,45^. We then studied RIP2 ubiquitination in the presence of mutant XIAPs in HEK 293 T cells, which showed that all tested XIAP-derived BIR2 mutations significantly impaired XIAP-mediated endogenous RIP2 ubiquitination (Fig. 6a). This suggests that BIR2-mutant XIAPs fail to ubiquitinate RIP2, leading to in NOD2 signaling malfunction.

We have previously shown that XIAP-ΔG204 unbound to RIP2 auto-ubiquitinated XIAP, causing XIAP degradation and deficiency^8^. We thus investigated the XIAP auto-ubiquitination at K31 of these mutant proteins using LC–MS/MS (Fig. 6b). The LC–MS/MS detected the specific enrichment of only the modified lysine-containing peptides of ubiquitinated mutant proteins. The presence of GG on the lysine side-chain represents a formerly ubiquitinated peptide that is cut out and quantified for XIAP auto-ubiquitination. Additionally, when XIAP is overexpressed with RIP2, three XIAP ubiquitination sites, K31, 168, and 297, were detected (Supplementary Fig. 4). Analyzing this data, our findings demonstrate that XIAP auto-ubiquitination at K31 is more dominant than K297 in most XIAP mutants. Furthermore, while it was confirmed that GG was attached to K168, the quantity of GG at K168 was so minute that it could not be quantified accurately. In accordance with co-IP data presented in Fig. 1a, XIAP-W173G exhibited less auto-ubiquitination patterns similar to that of XIAP WT (Fig. 6b) while the interaction between XIAP-W173G and RIP2 was noticed (Fig. 1a). In overall, LC–MS/MS analysis indicated that when XIAP-WT or mutants and RIP2 were overexpressed together, the mutant XIAP expression level was significantly reduced than that of XIAP-WT (Fig. 6C). It can be assumed that mutant XIAPs failed to bind to RIP2 and auto-ubiquitinated, thus degrading XIAP. One interesting point is that the expression level of W173G XIAP protein consistently remained low when cells were co-transfected with a ubiquitin-expressing plasmid (data not shown), indicating the necessity for further clarification regarding this mutant XIAP protein.

## Discussion

The *XIAP* gene was identified in 1996^46^, and its pathogenicity in a primary immunodeficiency in patients with a similar form of X-linked lymphoproliferative syndrome (XLP) was identified 10 years later^1^. Previously, XLP was thought to be caused by *SH2D1A* mutation, with phenotypes including hypogammaglobulinemia, hemophagocytic histiocytosis, splenomegaly, cytopenia, and hypogammaglobulinemia^47^. Later, XLP-like disorders caused by *XIAP* mutations were identified and named as XLP-2. XLP-2 phenotypes are slightly different from common XLP-1 phenotypes and most patients with XIAP deficiency showed colitis^48^. XIAP is more highlighted in a study reporting that 4% male patients with pediatric onset IBD have XIAP deficiency^2^. Currently, XIAP deficiency should be considered in patients suspected to have IBD with early onset or an refractory clinical course to conventional therapy including biologics^49^. Furthermore, in the era of exome sequencing for monogenic IBD^50^, the prevalence of XLP is expected to increase.

XIAP, cIAP1, and cIAP2 are the best-characterized proteins in the IAP family. These IAPs comprise three common Zn-binding BIR domains and a RING domain. Over 80 amino acids of BIR domains commonly constitute 4–5 α-helices, a three-stranded antiparallel β-sheet, and a Zn ion-containing core^51^. BIR2 and BIR3 are primarily responsible for inhibiting caspases, whereas XIAP is the only IAP inhibiting both apoptosis initiator (caspase-9) and effectors (caspase-3 and -7)^52^. Notably, the BIR2 domain of XIAP (residues 163–234) comprises a three-stranded antiparallel β-sheet surrounded by four α-helices that interact with RIP2 protein. This interaction is critical in controlling the NOD signaling, whose defect cause the hyperinflammation of mucosal homeostasis^1–5^. Approximately 100 *XIAP* PVs have been identified^3,48,49^. Two major genetic pathogeneses of XIAP deficiency have been uncovered: [1] the loss of protein expression by nonsense mutations, large deletion of exons, and splicing mutations; and [2] non- or dysfunctional protein owing to non-synonymous mutations. Non-synonymous mutations are frequently observed on BIR2 and RING domains. Non-synonymous genotype and phenotype are not correlated^14,15^. To our best knowledge, the specific role of non-synonymous mutations in RIP2 interaction has not been highlighted in terms of both structural analysis and molecular biology using proper quantitative metrics. Since defective interaction of XIAP mutants with RIP2 leading to defective NOD signaling causes the basic pathogenesis of colitis, understanding the altered phenomenon may be essential in studying refractory colitis with *XIAP* mutations.

**Figure 7:**
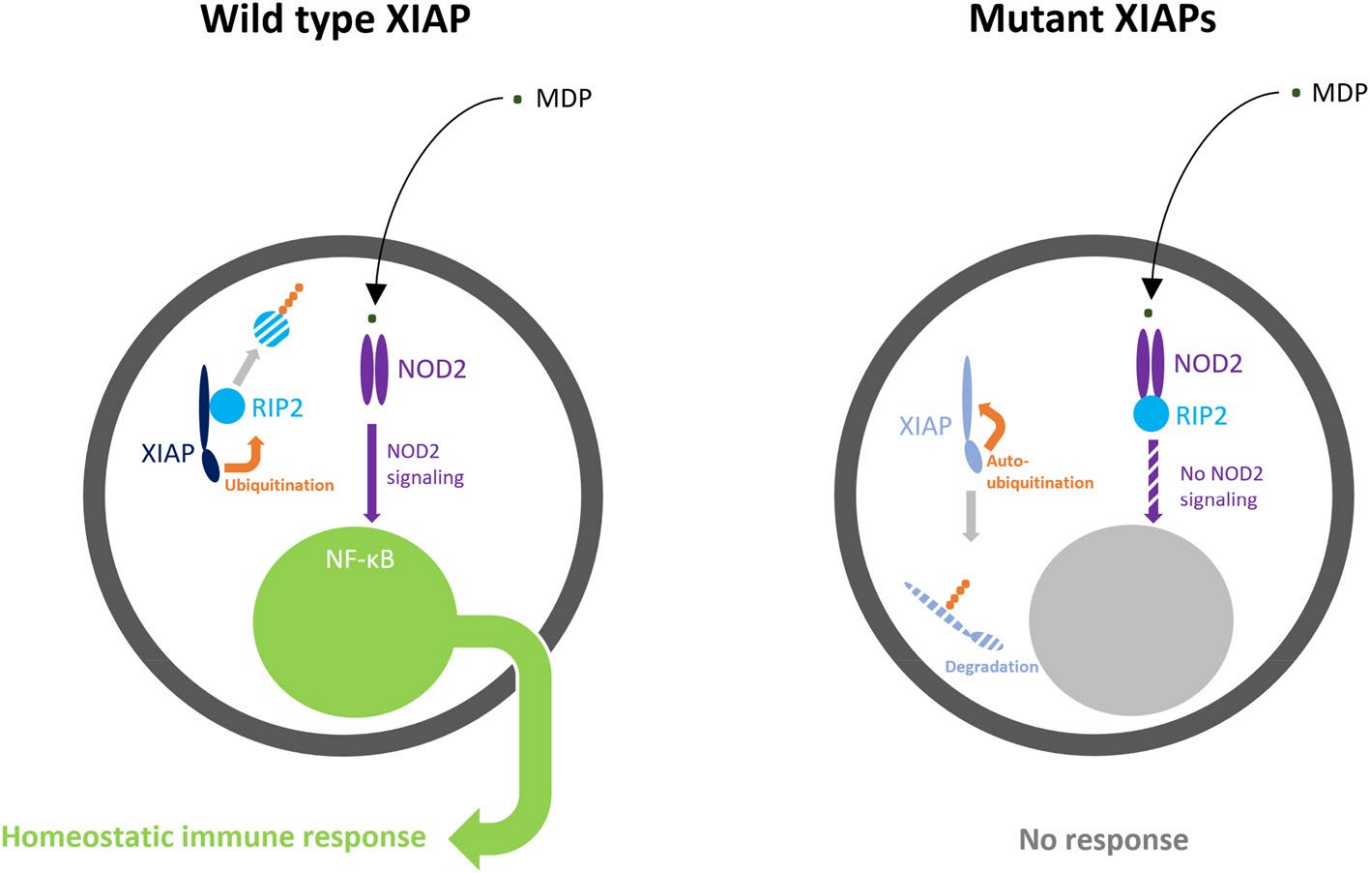
Pathogenic mechanisms of nonsynonymous mutant XIAP BIR2s. Our findings suggest a novel concept of a two-step mechanism in the development of XIAP deficiency caused by nonsynonymous pathogenic variants. The first step is a binding failure of mutant XIAPs to RIP2, resulting in an inability to interact with RIP2 and subsequently ubiquitinate RIP2. Given that NOD2 signaling can be activated in response to intracellular pathogens by ubiquitinating RIP2, a binding failure to RIP2 would be the first mechanism to evoke the XIAP-deficient phenomenon. The second step is the auto-ubiquitination of mutant XIAPs, which results in a relative deficiency of these mutant proteins. Traditionally, true XIAP deficiency is, in a strict sense, defined in cases of null variants, such as nonsense mutations, large insertions/deletions in exons, and critical splicing mutations. In cases of nonsynonymous pathogenic variants, previously unrecognized, mutant XIAPs also result in another type of XIAP protein deficiency, possibly contributing a synergistic impact on defective NOD2 signaling.

The messages from our analyses on non-synonymous *XIAP* PVs are clear. First, all non-synonymous PVs consistently do not bind to RIP2. Second, structural analysis showed that these mutations alter BIR2 structures such as hydrophobic core collapse, spatial rearrangement, and Zn-finger loss. Subsequently, two essential contact structures for binding RIP2, which are the two loops (loops 174–182 and 205–215), are unstable in holding RIP2. Third, XIAP mutants, which failed to bind RIP2, could not ubiquitinate RIP2 and enhanced auto-ubiquitination of XIAP mutants was noted. Subsequently, relative XIAP deficiency occurred owing to auto-ubiquitination and degradation. These findings constitute the initial succinct insights into the structural and molecular consequences of non-synonymous XIAP mutations. Overall, our findings suggest a novel concept of a two-step mechanism in the development of XIAP deficiency caused by nonsynonymous pathogenic variants (Fig. 7).

The characterization of these PVs provides potential therapeutic avenues in XIAP deficiency with BIR2 mutations. First, the function of non-synonymous mutant XIAPs is like that of null variants in the point of binding RIP2 and prevents RIP2 ubiquitination. Therefore, controlling XIAP behavior such as enhancing RIP2 binding using other molecules may not be helpful in targeting XIAP deficiency. Second, XIAPs with non-synonymous BIR2 mutations undergo auto-ubiquitination and subsequent protein deficiency. Non-synonymous mutation conditions cause not only dysfunction, but also loss of protein, and suggesting a potential therapy: protein replacement therapy.

This study has several limitations. First, we used N-terminally-fused GFP and RFP tags to evaluate XIAP and RIP2 localization, respectively, in HEK 293 T cells. Hence, the precise role and kinetics of the two pure proteins without GFP and RFP are unknown and C-terminally-fused tags were not tested in this study. Second, the HEK 293 T cell line has been widely utilized in the structural and functional analyses of XIAP proteins^5^. However, HEK 293 T cells do not accurately reflect the hematopoietic environments where NOD signaling is weighted in mucosal homeostasis. In addition, the human embryonic kidney cell-derived HEK 293 T cell line may express minimal XIAP-WT endogenously as XIAP is expressed ubiquitously^53^. Third, despite using substantial supercomputing resources, we did not calculate the binding free energy between XIAP mutants and RIP2. We believe that this is not essential in understanding the phenomenon of binding XIAPs mutant with RIP2. Fourth, benign variants of *XIAP* gene were not included as negative controls in our study. In a strict sense, using negative controls and positive controls is crucial for validating findings of testing PVs. Nevertheless, we meaningfully analyzed the impact of mutations on protein structure through consistent and precise analysis of not only local structural changes but also global structural changes.

In conclusion, this is the first structural and functional analysis on the non-synonymous XIAP BIR2 mutants, which have not often been discussed in detail. Non-synonymous XIAPs failed to bind to RIP2 owing to intra-structural instability and two unstable loop arms. Subsequently it resulted in a RIP2 ubiquitination failure and loss of protein deficiency by auto-ubiquitination of all XIAP mutants. These findings could benefit our understanding of the role of *XIAP* mutations in XIAP-deficient IBD, and hence future therapeutic strategies.

### Supplementary Information

Supplementary Information.

## Data Availability

The Processed data and images used in the analysis are available in the Supplemental Material file.
