# Supplementary material for: Understanding the molecular mechanism of pathogenic variants of BIR2 domain in XIAP-deficient inflammatory bowel disease
Source: Sci Rep. 2024 Jan 9;14:853. doi: 10.1038/s41598-023-50932-5 (PMC10774423; doi:10.1038/s41598-023-50932-5)

**Supplementary Information file for**

**“Understanding the molecular mechanism of pathogenic variants of BIR2 domain in XIAP-deficient inflammatory bowel disease”.**

- **Supplementary figure 1~4**
- **Link to the (1) Input parameter files, (2) Starting structures files used in molecular dynamics**
- **Uncropped whole blot images for the western blotting data in figure 1a, and 6a.**
- **Supplementary Tables 1, 2, and 3 provide the raw data corresponding to the graphical representations in Figure 1b, 6b, and 6c, respectively.**

**Supplementary figure 1~4**

**Supplementary figure 1. Representative positions of pathogenic variants within the XIAP BIR2 domain on the schematic diagram of mutant XIAP proteins** [reference sequence: NM_001167.3]. Currently, 14 specific loci have been linked to pathogenic variants in the BIR2 domain, as confirmed in clinically established cases of XIAP deficiency.^1-6^ Of them, eight loci had non-synonymous variants.


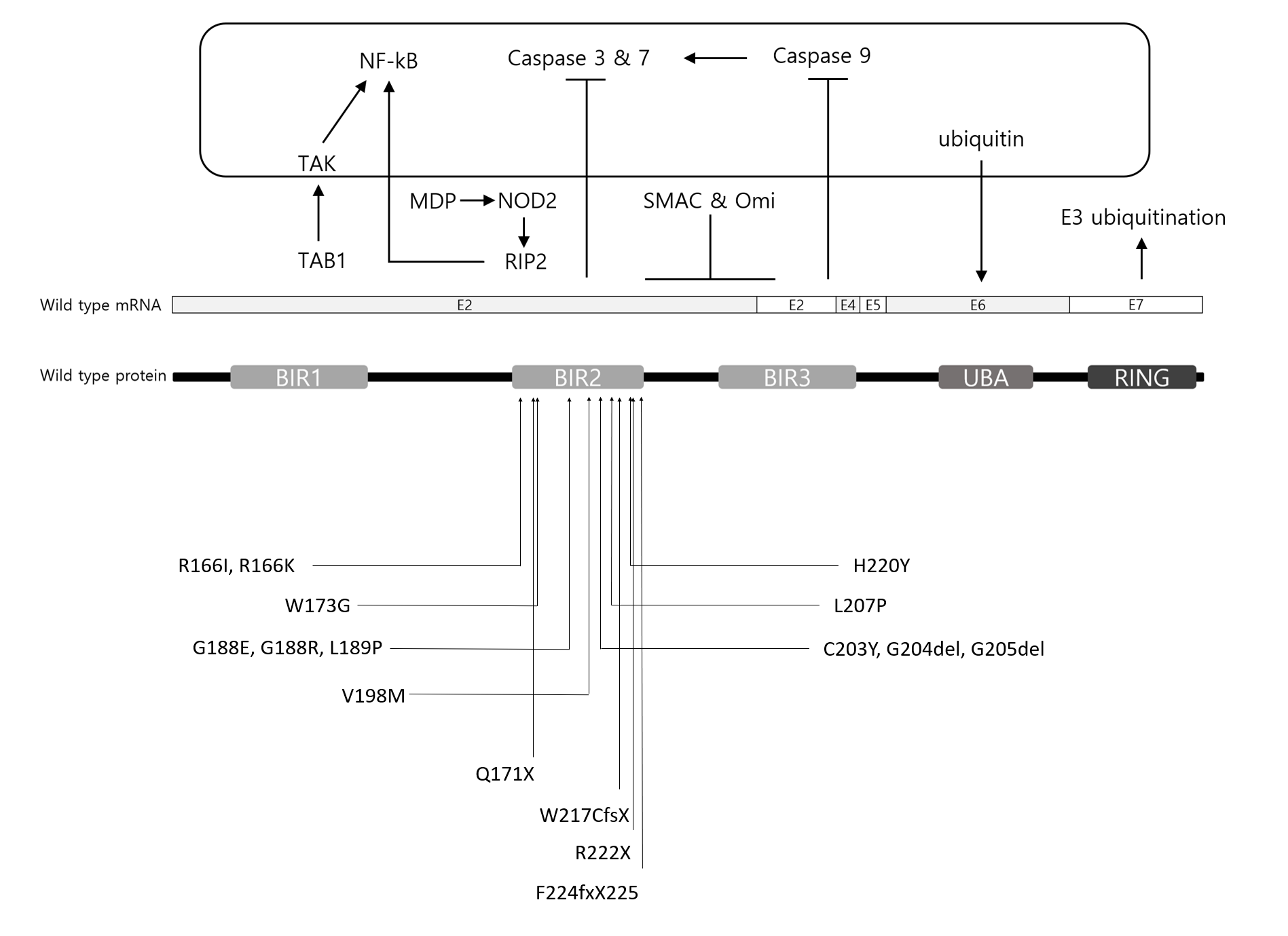


**Supplementary Figure 2. Histogram of the minimum distance between the 198^th^ and 207^th^ amino acids, measured at the initial and final 500ns simulation.** (a-b) During the simulation of the WT, the distance between V198 and L207 is closely located. Due to the G188E mutation, (c) the distance between V198 and L207 increases at the Early time of the simulation, and (d) the probability of the long-distance distribution becomes larger at the end of the simulation. In the case of L207P, (e) a small number of ensembles with a long distance between V198 and P207 are found early in the simulation time, and (f) the number of long-distance ensembles increases with time.

**
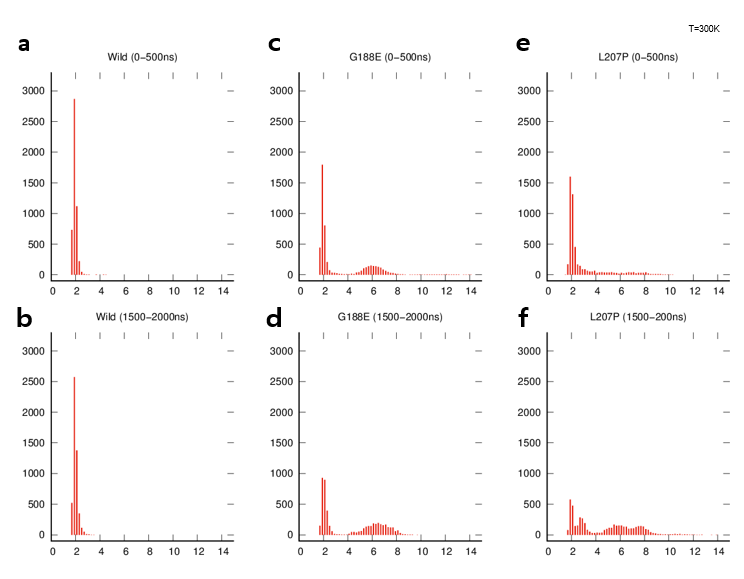
**

**Supplementary Figure 3. Effect of H220Y mutation on Zn-finger.** (a) Baculovirus IAP repeat 2 domain of X-linked inhibitor of apoptosis protein (XIAP BIR2) Zn-finger structure. Zn ion indicated using a gray ball. (b) H220Y mutation-mediated structural changes causing Zn loss. (c) The root mean square fluctuation (RMSF) of wild-type XIAP BIR2 (XIAP BIR2-WT) and -H200Y. Zn-fingers and mutation point are indicated in yellow and red arrows, respectively, 205–215 loop region highlighted in blue. (d) The root mean square deviation (RMSD) of XIAP BIR2-H220Y mutant hydrophobic core.

**
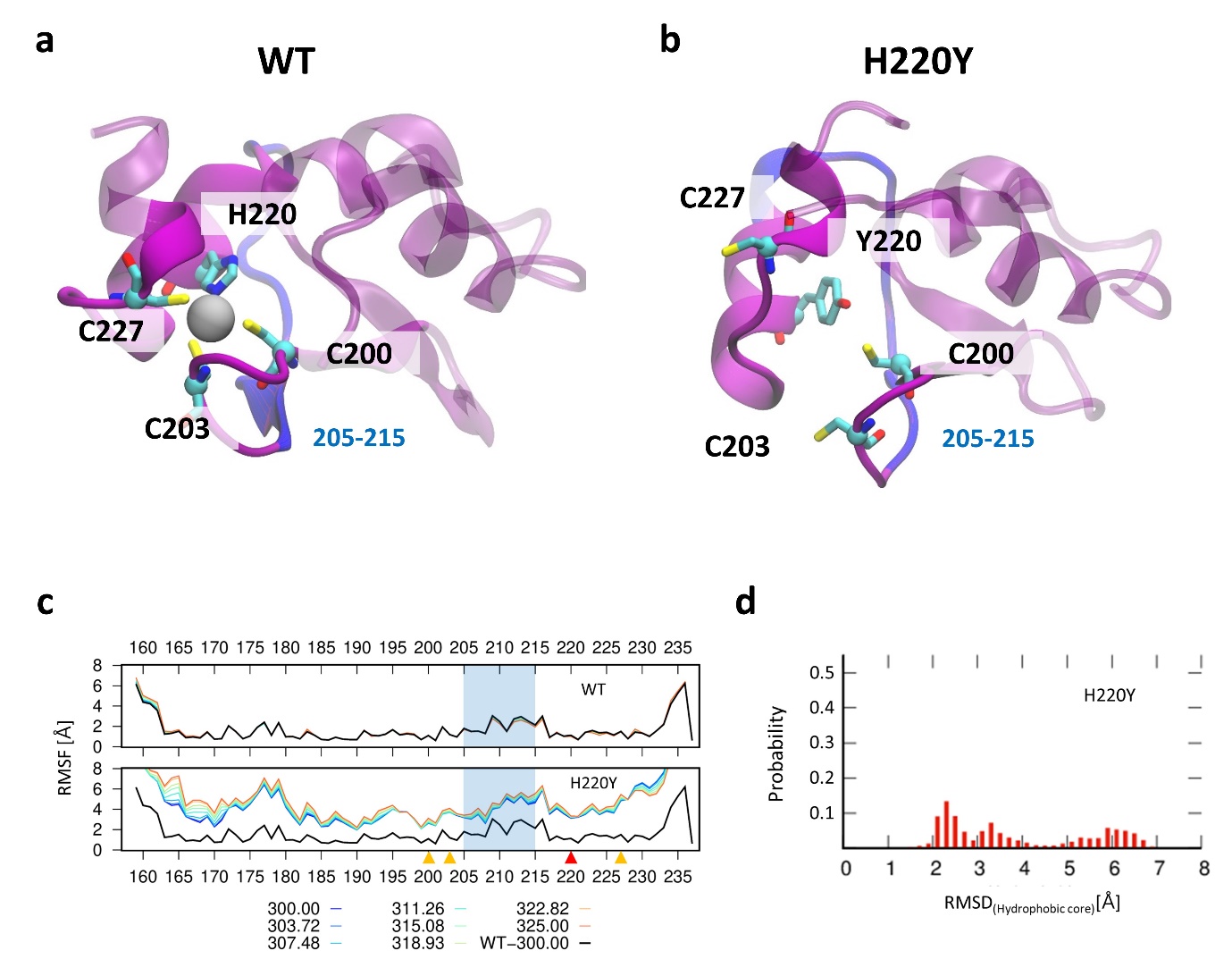
**

**Supplementary figure 4. Characterization of X-linked inhibitor of apoptosis protein (XIAP) ubiquitination sites by mass spectroscopy.** (a) Amino acid sequence of XIAP. Possible ubiquitination sites are indicated in red font. (b) Di**-**glycine-lysine (GG) remnants after trypsin digestion indicate possible ubiquitination sites. Note that K168 ubiquitination was confirmed by the described method, however quantitatively assessing the extent of ubiquitination at this site was not feasible.


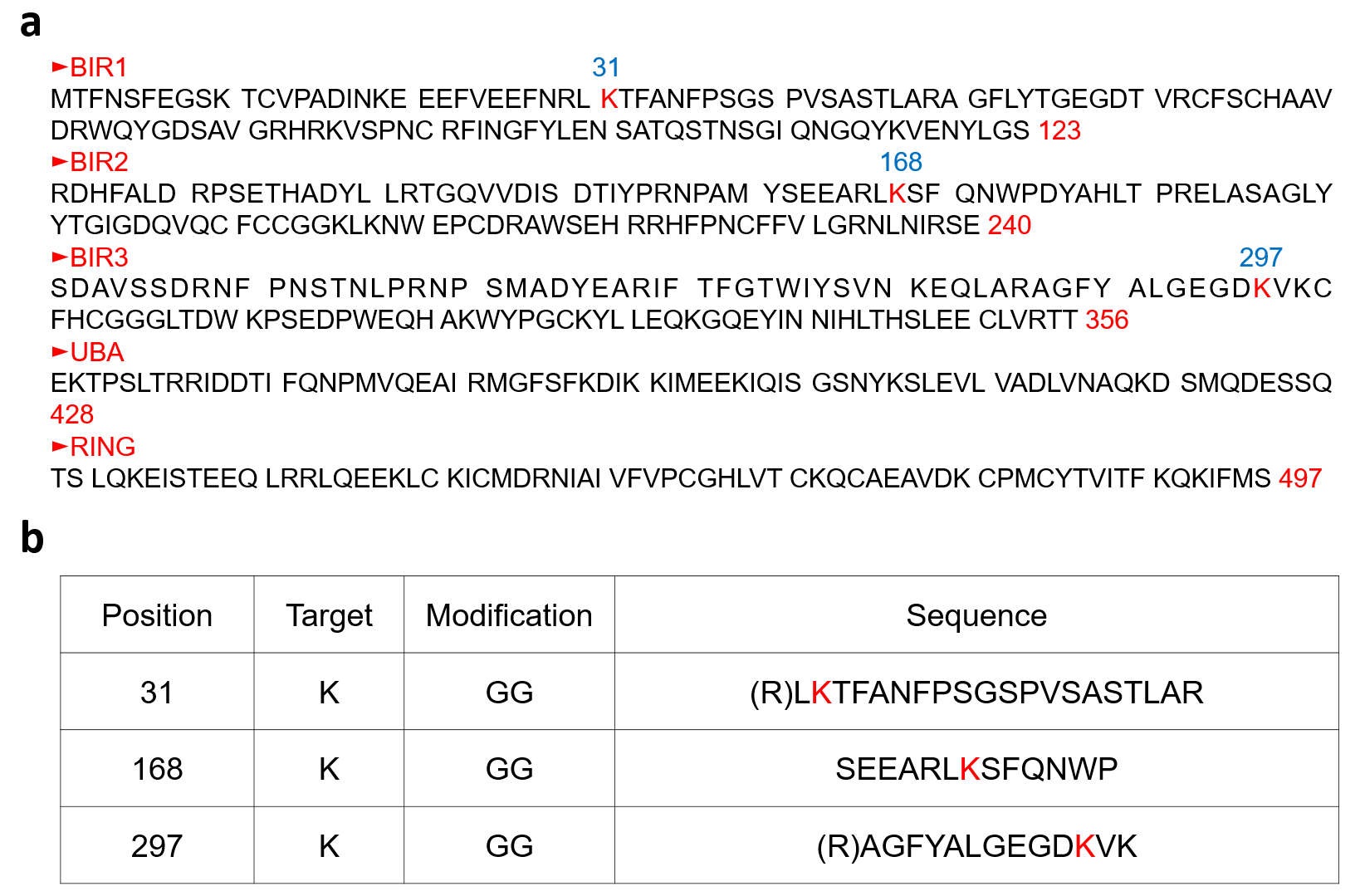


**Link to the (1) Input parameter files, (2) Starting structures files used in molecular dynamics**

- **The following files and data are stored in google drive**
- **Cloud URL :**

[**https://drive.google.com/drive/folders/18jwGrfkIcutMEJ1aKRsMsPqOyrkcGQVL?usp=sharing**](https://drive.google.com/drive/folders/18jwGrfkIcutMEJ1aKRsMsPqOyrkcGQVL?usp=sharing)

- **List of files**

**1. Input parameter Files**

**- 00_min.in, 01_heat.in, 02_eq.in, REMD.in**

**2. Initial structure and parameter files**

**-00_Wild.pdb, 00_Wild.rst7, 00_Wild.prmtop**

**-01_H220Y.pdb, 01_H220Y.rst7, 01_H220Y.prmtop**

**-02_R166I.pdb, 02_R166I.rst7, 02_R166I.prmtop**

**-03_R166K.pdb, 03_R166K.rst7, 03_R166K.prmtop**

**-04_G188E.pdb, 04_G188E.rst7, 04_G188E.prmtop**

**-05_W173G.pdb, 05_W173G.rst7, 05_W173G.prmtop**

**-06_L189P.pdb, 06_L189P.rst7, 06_L189P.prmtop**

**-07_V198M.pdb, 07_V198M.rst7, 07_V198M.prmtop**

**-08_L207P.pdb, 08_L207P.rst7, 08_L207P.prmtop**

**References**

1 Zeissig, Y. *et al.* XIAP variants in male Crohn's disease. *Gut* **64**, 66-76 (2015).

2 Ashton, J. J. *et al.* Identification of Variants in Genes Associated with Single-gene Inflammatory Bowel Disease by Whole-exome Sequencing. *Inflammatory bowel diseases* **22**, 2317-2327 (2016).

3 Aguilar, C. *et al.* Characterization of Crohn disease in X-linked inhibitor of apoptosis-deficient male patients and female symptomatic carriers. *J Allergy Clin Immunol* **134**, 1131-1141.e1139 (2014).

4 Chang, I. *et al.* Interpretation of XIAP Variants of Uncertain Significance in Paediatric Patients with Refractory Crohn's Disease. *Journal of Crohn's & colitis* **15**, 1291-1304 (2021).

5 Pedersen, J., LaCasse, E. C., Seidelin, J. B., Coskun, M. & Nielsen, O. H. Inhibitors of apoptosis (IAPs) regulate intestinal immunity and inflammatory bowel disease (IBD) inflammation. *Trends Mol Med* **20**, 652-665 (2014).

6 Nielsen, O. H. & LaCasse, E. C. How genetic testing can lead to targeted management of XIAP deficiency-related inflammatory bowel disease. *Genet Med* **19**, 133-143 (2017).

**Uncropped blot images for the western blotting data in figure 1a, and 6a.**

**In all preparation procedures of Western blotting, the blots were cut before antibody hybridization.**

Fig. 1a


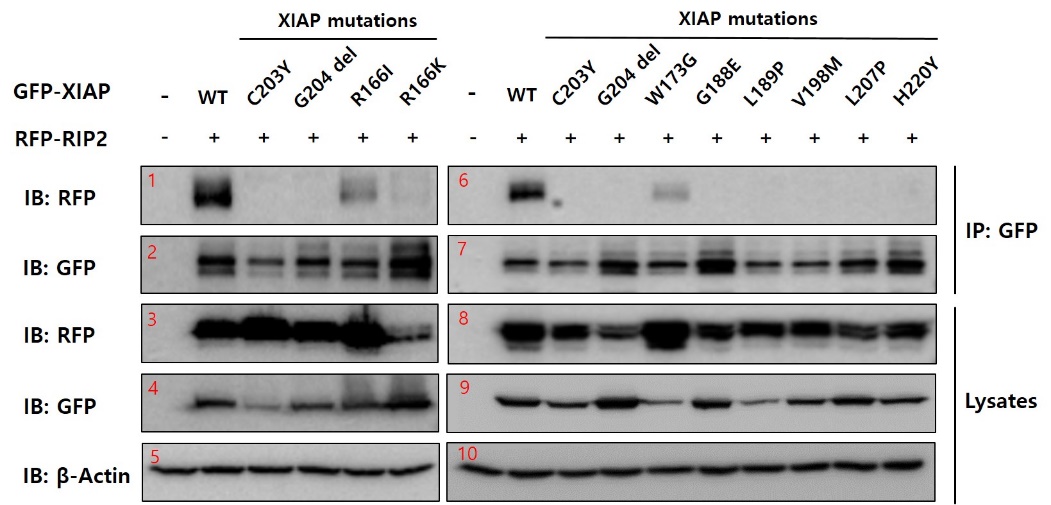


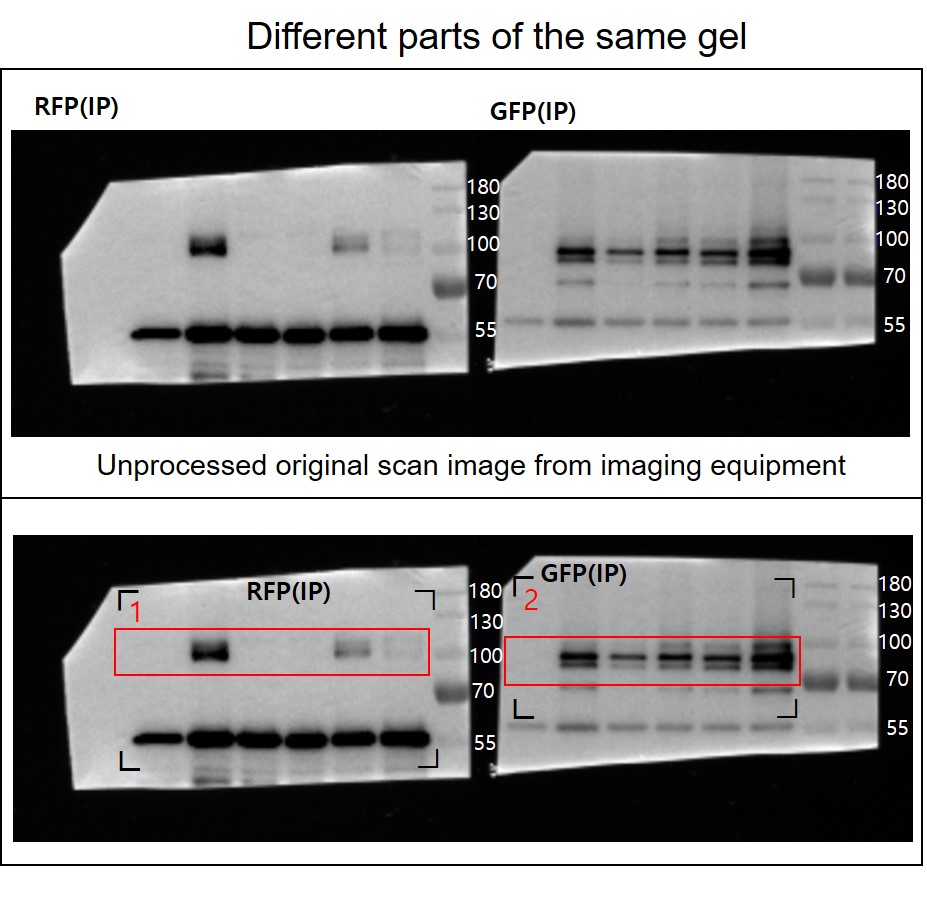


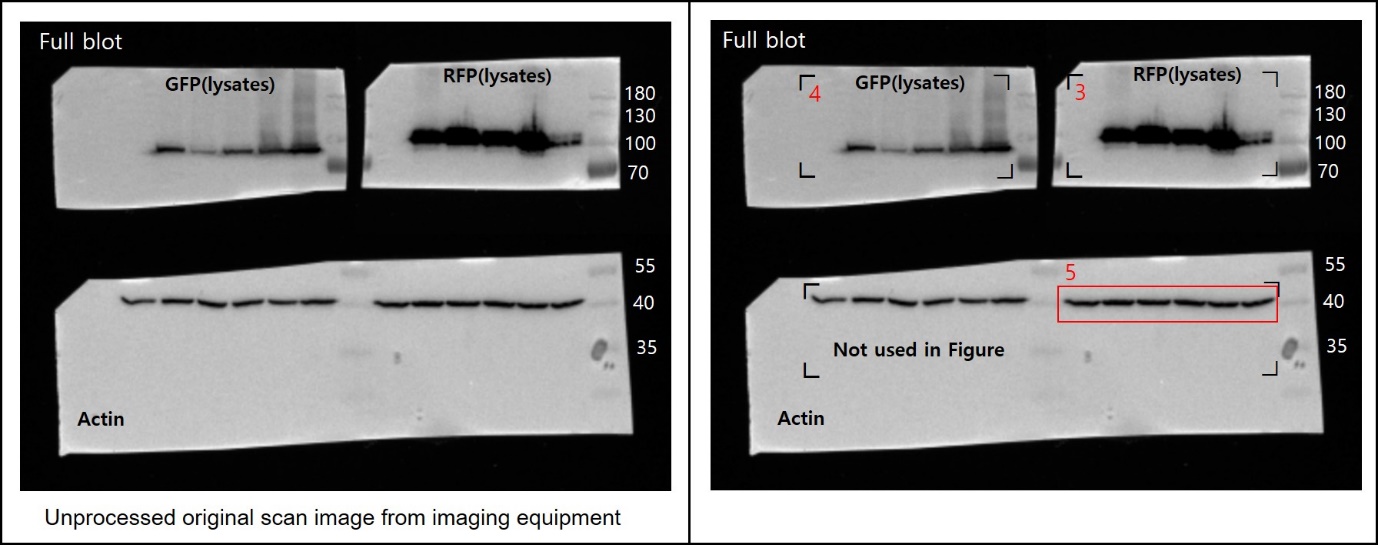


Fig 1a

*Polyvinylidene Fluoride (*PVDF), containing the protein bands were routinely cropped before hybridization. Specifically, cut below 70 kDa to distinguish it from Actin (42 kDa). Cells were transfected with 1 μg of EGFP-XIAP and 3 μg of RFP-RIP2, as RIP2 is degraded by XIAP. RIP2 exhibited strong expression. The image above represents a full-length membrane cut prior to hybridization with an antibody. There are two lines of Actin; however, only one is incorporated into the figure and highlighted with a red box. In the picture below, bands outside the red box are non-specific bands.

Lane 1, empty vector

Lane 2, GFP-XIAP WT+ RFP-RIP2

Lane 3, GFP-XIAP C203Y+ RFP-RIP2

Lane 4, GFP-XIAP G204del+ RFP-RIP2

Lane 5, GFP-XIAP R166I+ RFP-RIP2

Lane 6, GFP-XIAP R166K+ RFP-RIP2


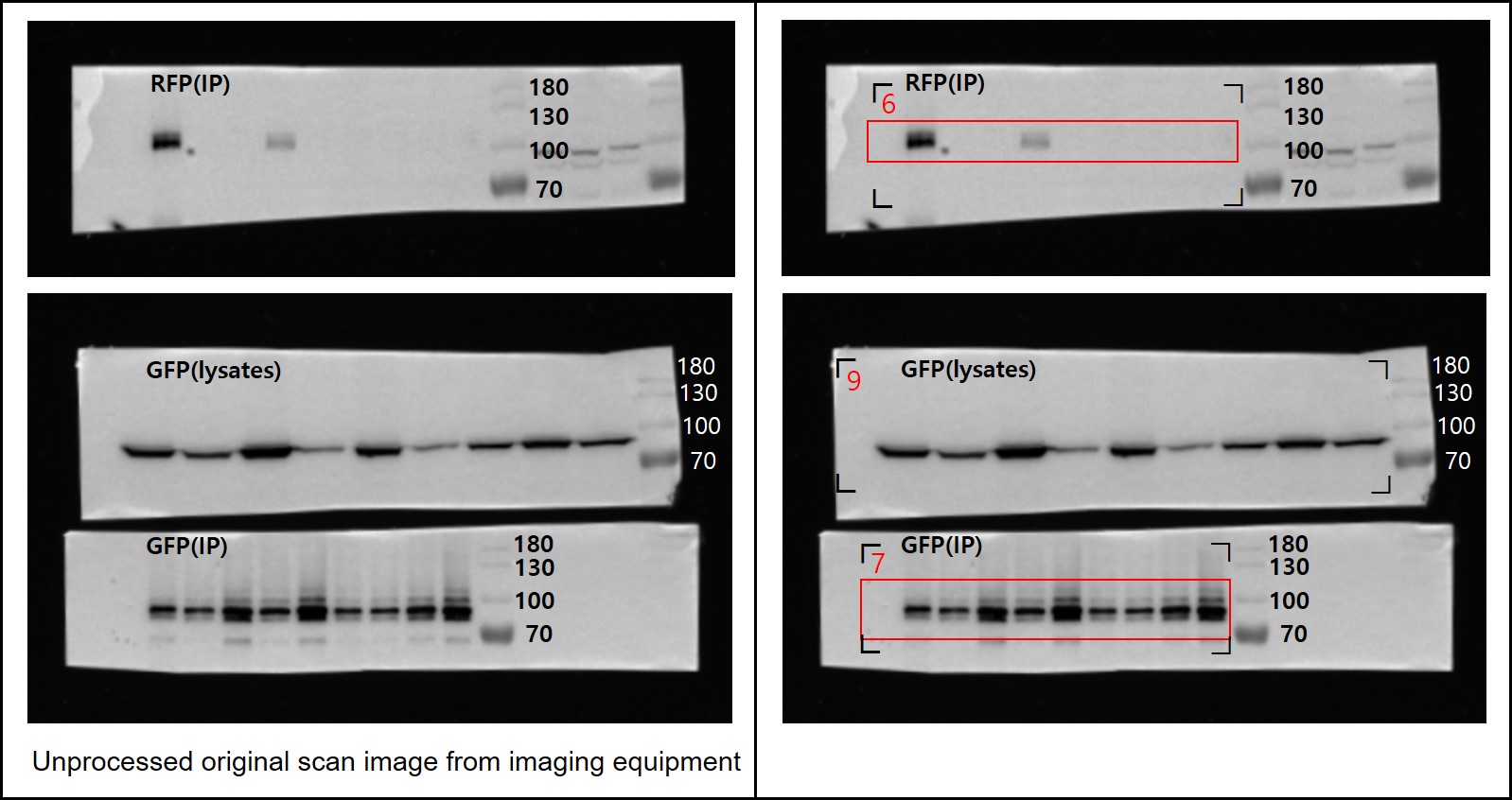


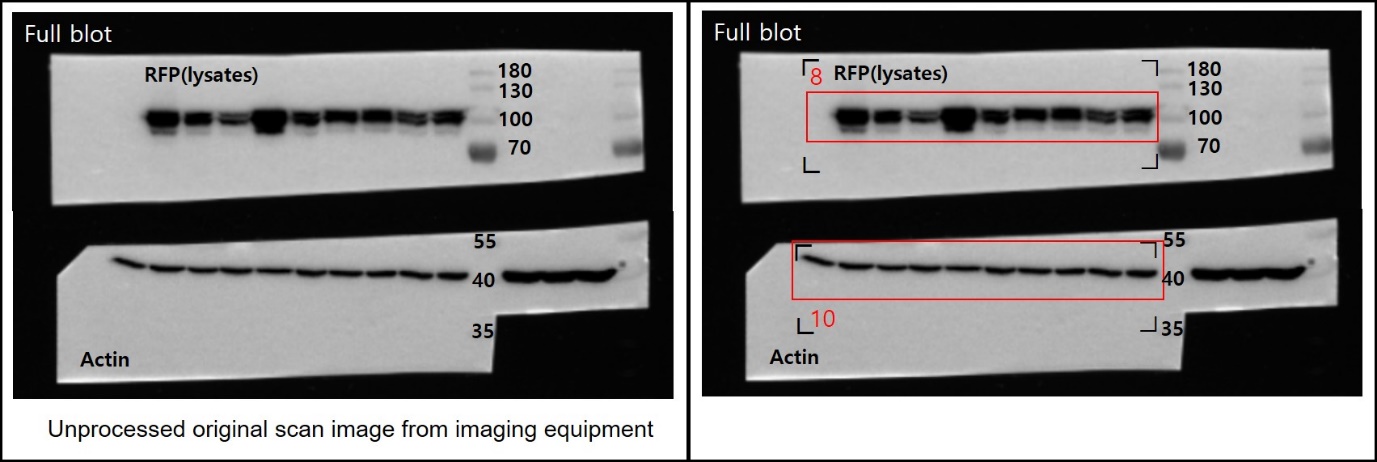


Continuing on Fig 1a

In Western experiments, a combination of 12 and 15 wells was employed. All bands in the 12 wells are entirely depicted in the figure, while only the left band in the 15 wells is illustrated in the figure (highlighted by the red box), and the right band is utilized in other experiments, serving no purpose as a reference in this context.

Lane 1, empty vector

Lane 2, GFP-XIAP WT+ RFP-RIP2

Lane 3, GFP-XIAP C203Y+ RFP-RIP2

Lane 4, GFP-XIAP G204del+ RFP-RIP2

Lane 5, GFP-XIAP W173G+ RFP-RIP2

Lane 6, GFP-XIAP G188E+ RFP-RIP2

Lane 7, GFP-XIAP L189P+ RFP-RIP2

Lane 8, GFP-XIAP V198M+ RFP-RIP2

Lane 9, GFP-XIAP L207P+ RFP-RIP2

Lane 10, GFP-XIAP H220Y+ RFP-RIP2

Fig. 6a


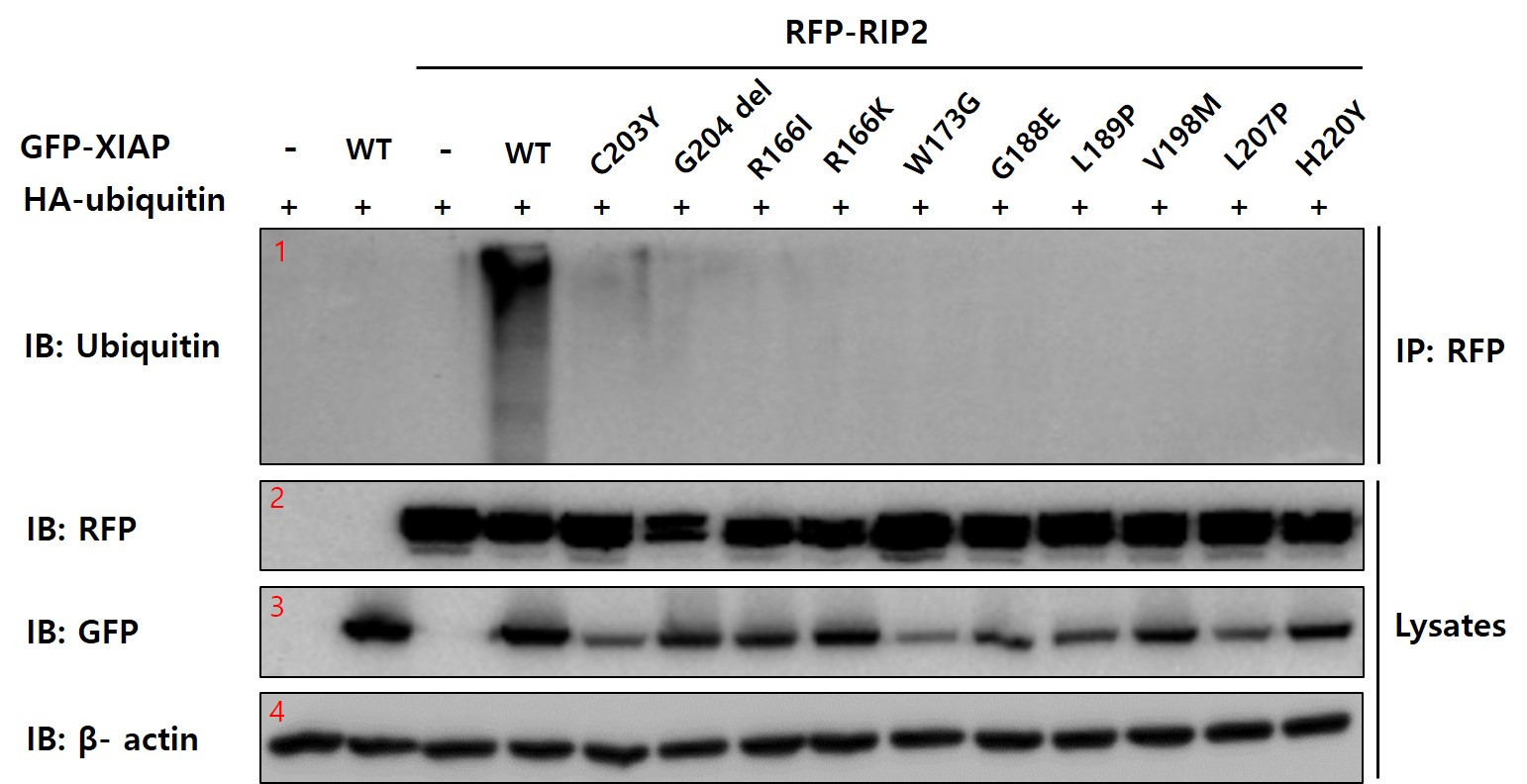


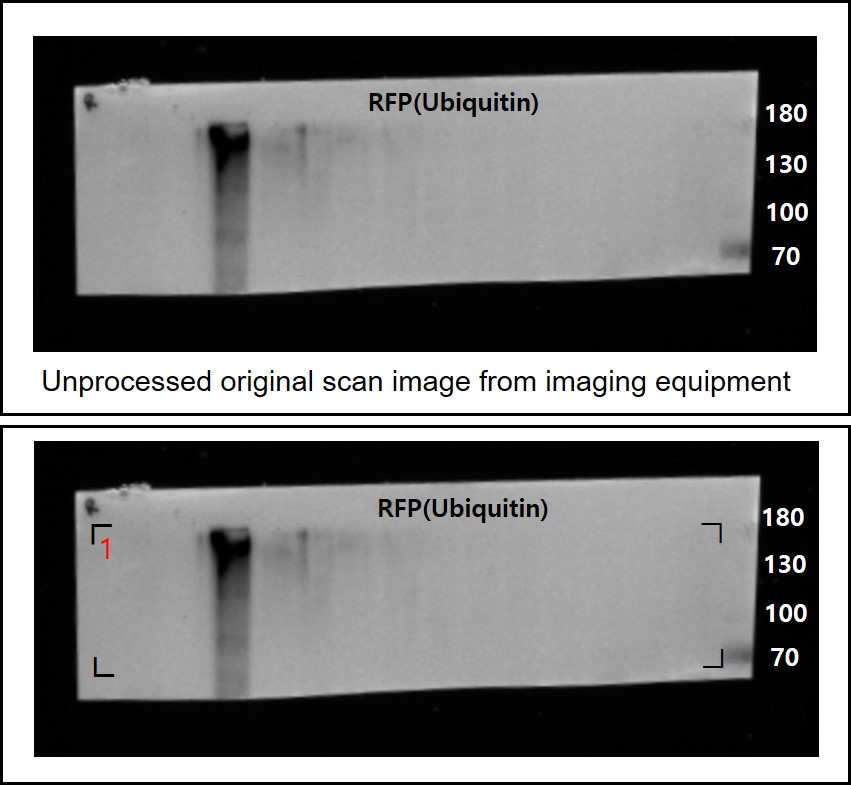


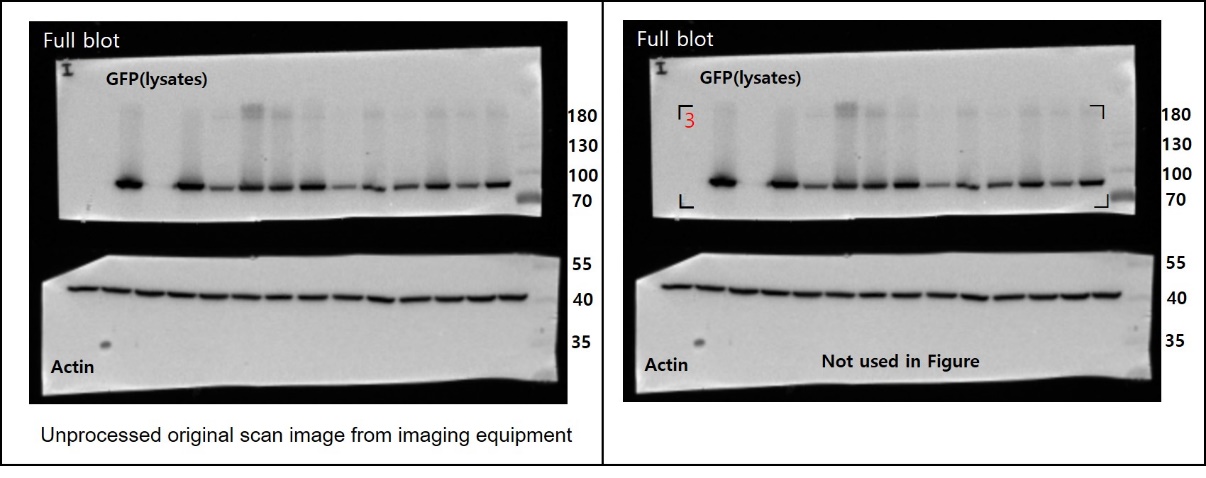


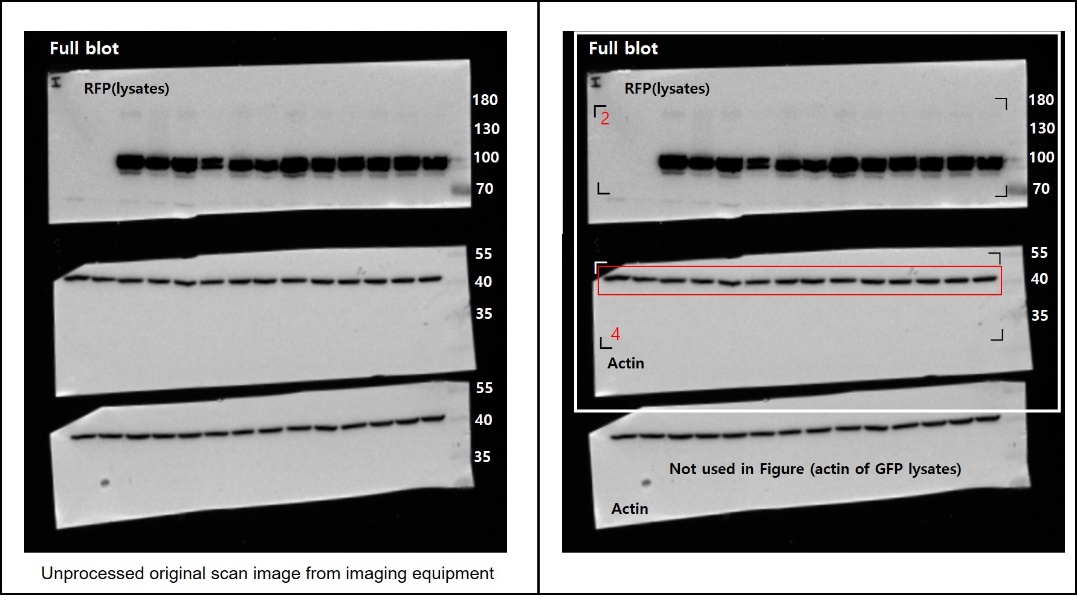


Fig 6a

*Polyvinylidene Fluoride (*PVDF), containing the protein bands were routinely cropped before hybridization. Specifically, cut below 70 kDa to distinguish it from Actin (42 kDa). Cells were transfected with 1 μg of EGFP-XIAP and 3 μg of RFP-RIP2, as RIP2 is degraded by XIAP. RIP2 exhibited strong expression. The white box signifies a band detected on one membrane. There are two lines of Actin; however, only one is incorporated into the figure and highlighted with a red box.

Lane 1, empty vector

Lane 2, GFP-XIAP WT

Lane 3, RFP-RIP2

Lane 4, GFP-XIAP WT+ RFP-RIP2

Lane 5, GFP-XIAP C203Y+ RFP-RIP2

Lane 6, GFP-XIAP G204del+ RFP-RIP2

Lane 7, GFP-XIAP R166I+ RFP-RIP2

Lane 8, GFP-XIAP R166K+ RFP-RIP2

Lane 9, GFP-XIAP W173G+ RFP-RIP2

Lane 10, GFP-XIAP G188E+ RFP-RIP2

Lane 11, GFP-XIAP L189P+ RFP-RIP2

Lane 12, GFP-XIAP V198M+ RFP-RIP2

Lane 13, GFP-XIAP L207P+ RFP-RIP2,

Lane 14, GFP-XIAP H220Y+ RFP-RIP2

**Supplementary Tables 1, 2, and 3 provide the raw data corresponding to the graphical representations in Figure 1b, 6b, and 6c, respectively.**

**Table 1. Related to Figure 1b.** **Correlation coefficient values between GFP-XIAP and RFP-RIP2 in FCCS**


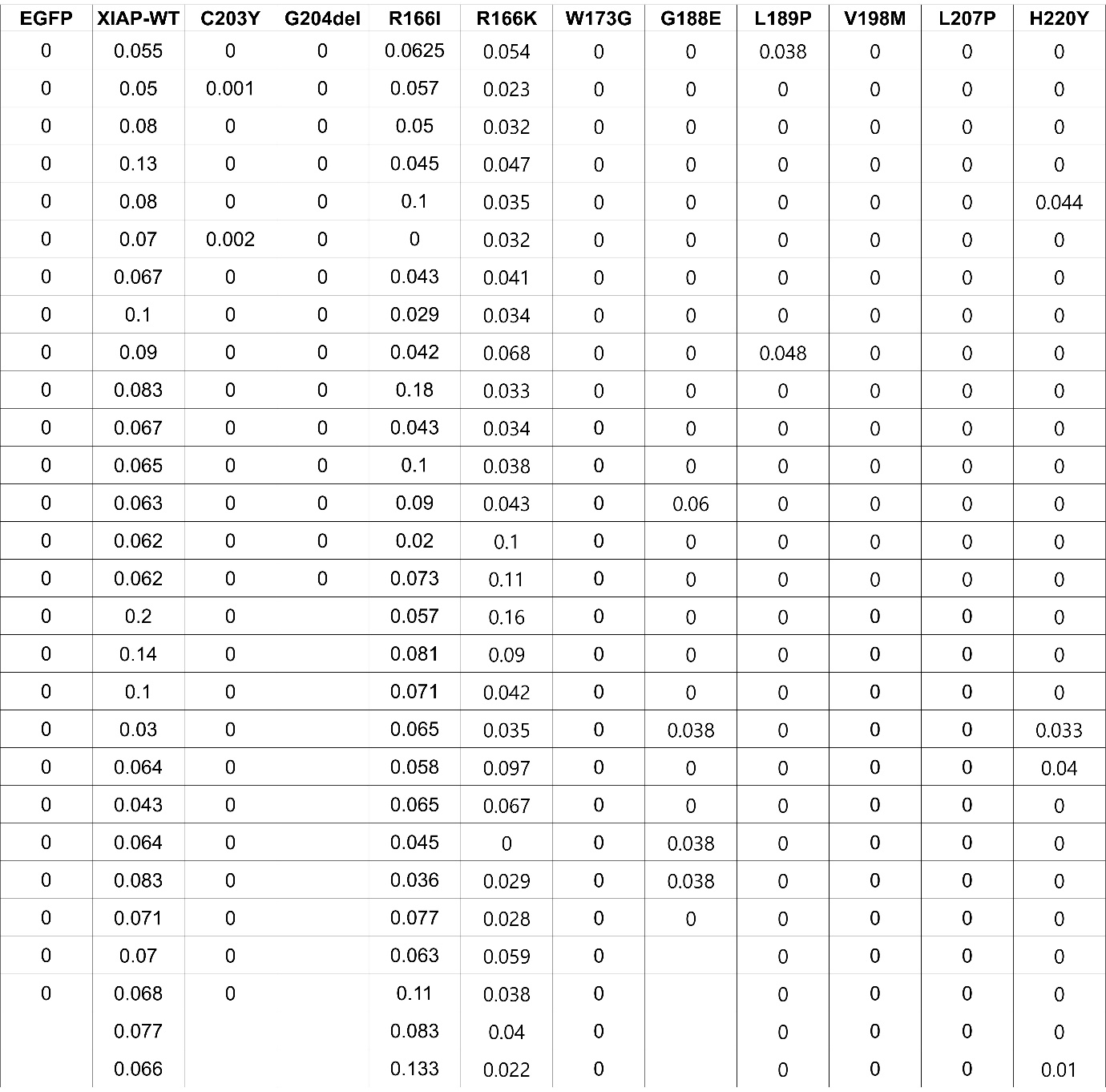


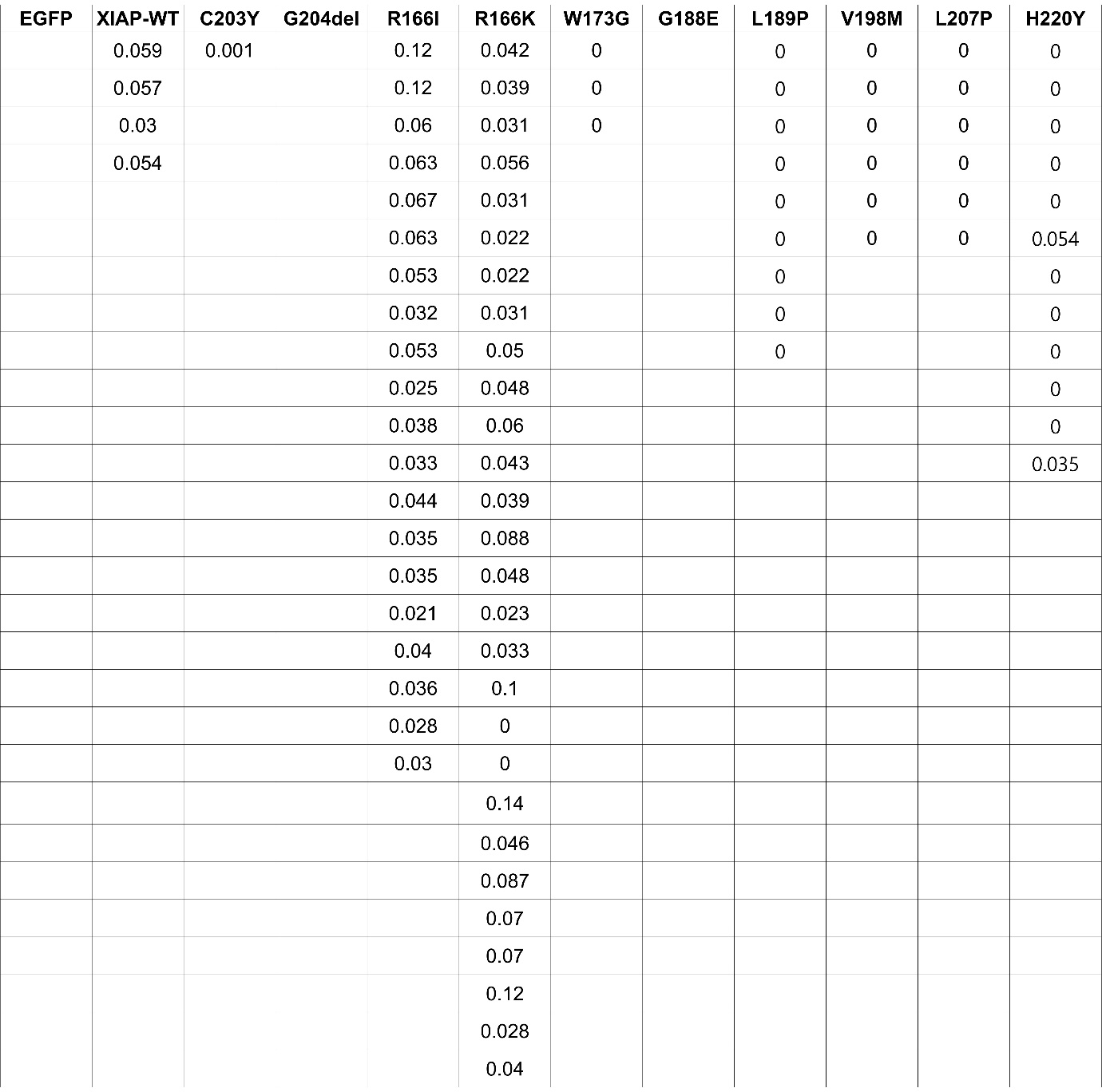


**
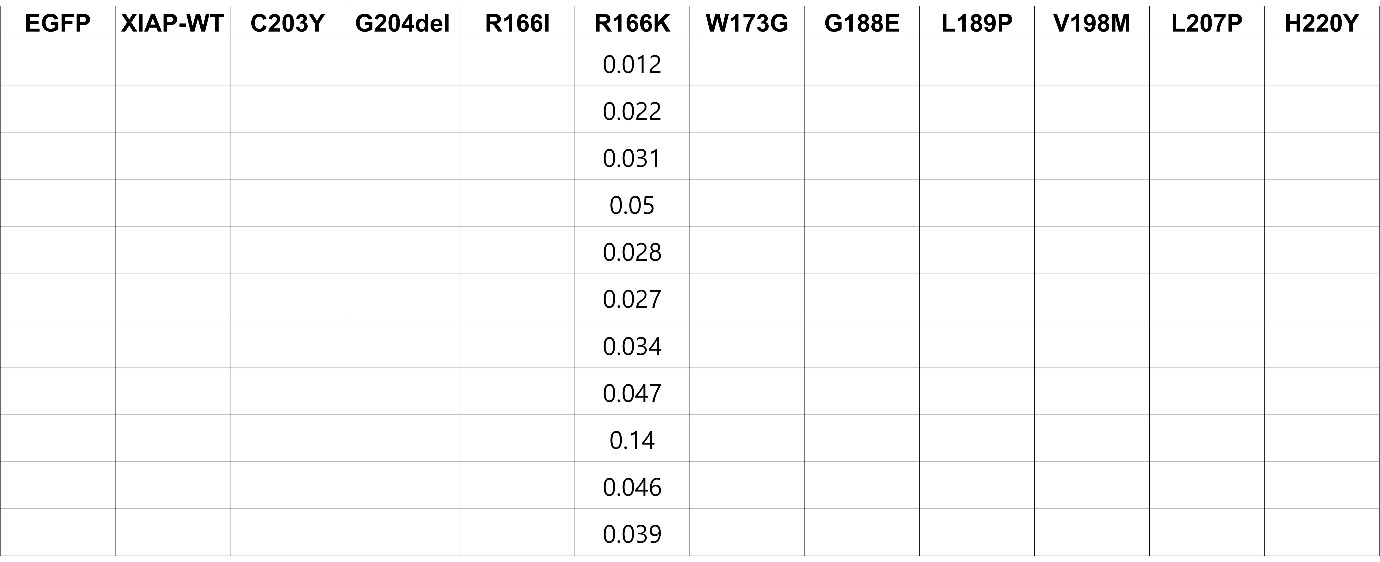
**

**Table 2. Related to Figure 6b. Label-free quantities of each di-glycine-attached XIAP**

**a. Analyzed quantity of di-glycine-attached XIAP peptide**
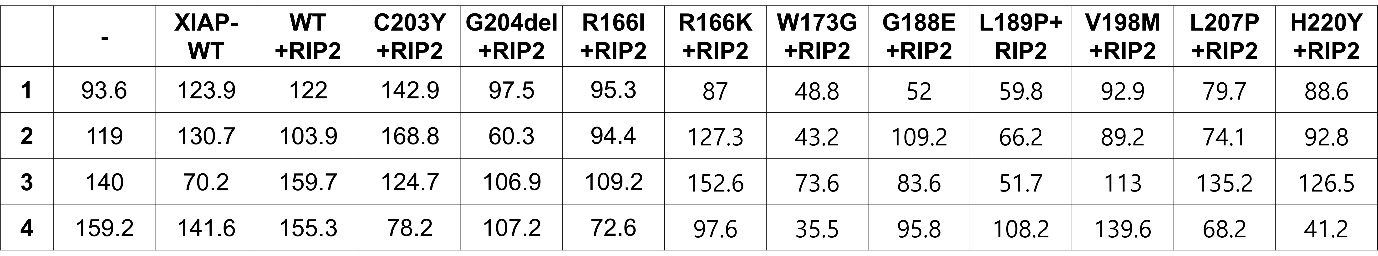


**b. Di-glycine-attached peptide/XIAP expression value**


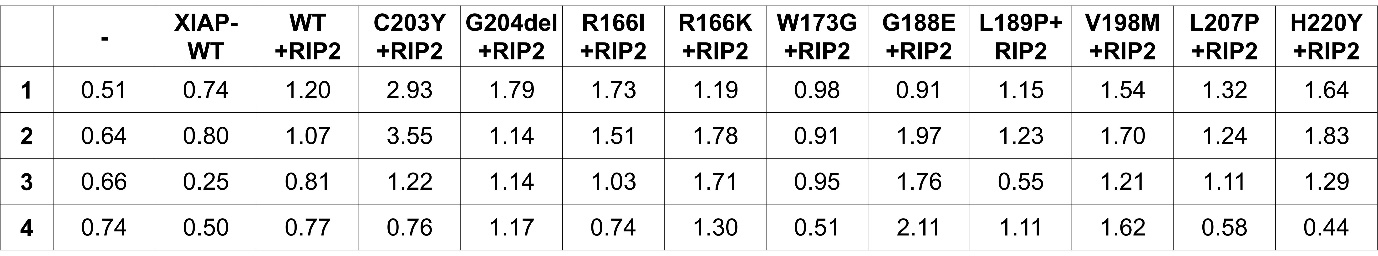


**Table 3. Related to Figure 6c**. **Total amount of XIAP expression in LC-MS/MS**


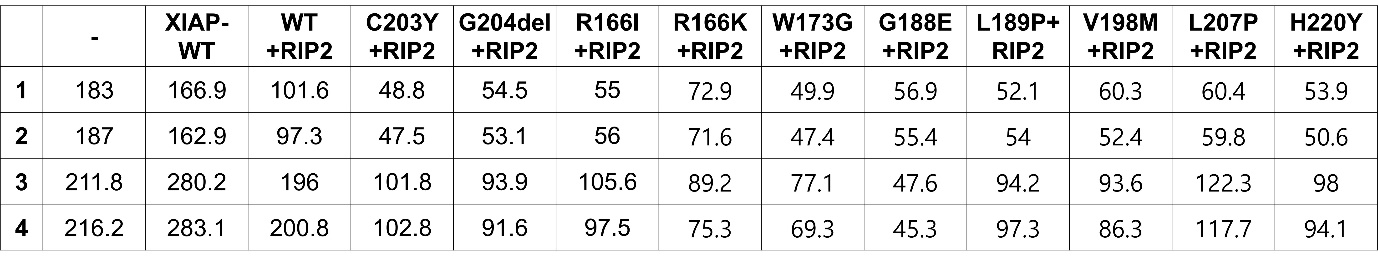

Supplement: Supplementary file 1 — Supplementary Information. [file 41598_2023_50932_MOESM1_ESM.docx]
